# Mycobiome Diversity of the Cave Church of Sts. Peter and Paul in Serbia—Risk Assessment Implication for the Conservation of Rare Cavern Habitat Housing a Peculiar Fresco Painting

**DOI:** 10.3390/jof8121263

**Published:** 2022-11-30

**Authors:** Milica Ljaljević Grbić, Ivica Dimkić, Željko Savković, Miloš Stupar, Aleksandar Knežević, Aleksa Jelikić, Nikola Unković

**Affiliations:** 1 Faculty of Biology, University of Belgrade, Studentski trg 16, 11000 Belgrade, Serbia; 2 Institute for the Protection of Cultural Monuments of Serbia—Belgrade, Radoslava Grujića 11, 11000 Belgrade, Serbia

**Keywords:** biodeterioration, integrative methods, mycobiota, sacral ambient, wall painting

## Abstract

The mycobiome of the cave Church of Sts. Peter and Paul, housing the peculiar fresco painting of “The Bald-headed Jesus”, was analyzed via culture-dependent and -independent methods. Salt efflorescence, colored patinas, and biofilm, as well as biopitting, discolorations, and fruiting bodies of wood-decay fungi were observed on surfaces within the church. Microscopic analyses showed an abundance of fungal structures, i.e., conidiophores, conidia, chlamydospores, and ascospores. The estimated values of the contamination classified all surfaces as the “Danger zone”. A total of 24 fungi from 17 genera were determined as part of the culturable mycobiome, with a dominance of *Ascomycota* of genera *Penicillium*. Biodegradative profiles analyzed via plate assays demonstrated positive reactions for 16 isolates: most commonly acid production (8), followed by pigment production and ligninolytic activity (6), protein degradation (5), cellulolytic activity (3) and carbonate dissolution (2). Metabarcoding analysis showed a dominance of *Ascomycota* in all samples (79.9–99.7%), with high relative abundance documented for *Hypoxylon fuscopurpureum* on the iconostasis and unclassified *Mycosphaerellaceae* family within order *Capnodiales* on fresco and stone, as well as moderate relative abundance for unclassified *Dothideomycetes*, *Botryolepraria lesdainii*, *Verrucaria* sp. and *Cladosporium* sp. on stone walls. The used set of integrative methods pointed out species of genus *Neodevriesia* and *H. fuscopurpureum* as the main deteriogenic agents of fresco and iconostasis surfaces, respectively.

## 1. Introduction

Since the dawn of humankind, traced back to the paleolithic ages, caves were used as primordial human shelters, as well as the first genuine places for artistic expression, as shrines, oracles, and places for magic and religious rituals. These subterranean sites were also known in Christian tradition to be repurposed as churches and sanctuaries by the construction of chapels and shrines at the cave entrances [1]. This was the case with the Cave Church of Üzümlü in Cappadocia, Turkey; the Crypt of the Original Sin in Matera; and the “Sant’Angelo in Criptis” cave church in Apulia, Italy; as well as Corbii de Piatră cave church in Argeș, Romania, to name the few [2,3,4,5]. In the last decades, due to remarkable geological features, usually earning them a status of a geoheritage site, and great cultural and spiritual significance, many cave churches have become tourist attractions. Because of their importance as witnesses of past communities and happenings, cave churches are also becoming milestones in landscape management, with local authorities aiming to preserve them to maintain both the local tradition and environment [5].

Due to specific environmental conditions, stone surfaces and fresco paintings decorating walls of lithic indoor environments are exposed to biodeterioration phenomena. It is characterized by a distinct phenomenology of alterations that can have spatial variations among the different areas of the walls [4]. Undesirable alterations are induced by different microbial communities that are part of cave biodiversity. Many of them coexist, interact with each other, and are embedded in extracellular polymeric substances, i.e., three-dimensional dynamic structures known as biofilm that establish on wall surfaces in contact with air [6]. Among various microorganisms (such as bacteria, cyanobacteria, algae, and fungi) that constitute a biofilm or are causative agents of various other alterations, microscopic fungi are usually considered the primary causes of deterioration of fresco paintings and other works of art found within (iconostasis, sacral relics, etc.) [7]. Within the caves, fungi can colonize a variety of substrata such as vermiculations on cave walls, cave sediments, carcasses, and bat guano, to name a few [8]. Cave stone walls made of limestone have low compressive strength and hardness and are highly bioreceptive and porose. Hence, they are prone to fungal colonization and later biodeterioration [9]. Recent studies confirmed that fungi can colonize limestone materials and cause deterioration via chromatic alterations, stone dissolution, and calcium oxalate formation [10,11]. Fungal propagules in caves could be dispersed by air and via visitors, especially in the caves of touristic and cultural heritage significance, such as cave churches, where they represent potential biodeterioration risk for objects within [12]. This is due to them being ubiquitous and cosmopolitan, with pronounced enzymatic activity and the ability to grow on substrates with low water activity [13]. As such, they can degrade all the organic and inorganic components of fresco paintings resulting in aesthetically unacceptable changes. In numerous cultivation-based studies conducted in the last few decades, as well as a limited number of studies based on metabarcoding analysis of unculturable samples, mycobiome of fresco paintings within historic buildings, churches and hypogea were mainly dominated with *Ascomycota*, while *Zygomycota* and *Basidiomycota* were less frequent on frescoes, but abundant in the surrounding environment [14,15,16,17,18]. In the case of cave churches, this diversity can be further increased by touristic activities that favor the dispersion and introduction of new fungi, enrich the environment with organic and inorganic leftovers, change pristine climate (e.g., temperature, the concentration of carbon dioxide, humidity, etc.), requiring additional and careful consideration and research [1]. Several studies have suggested combining culture-dependent and -independent approaches to obtain a sounder conclusion regarding the total microbial communities in the environment [18].

To the best of our knowledge, up to date, the literature on culturable and total mycobiome, as well as fungal-induced deterioration of unique geoheritage sites such as cave churches is still scarce. Having that in mind, the principal purpose of the study was to analyze fungal communities involved in the deterioration of limestone walls, fresco, and iconostasis surfaces within the rare subterranean habitat of Cave Church of Sts. Peter and Paul in village Rsovci (Serbia), evaluate their ecological features and deteriogenic ability, so adequate management and monitoring actions can take place, and a conservation plan and intervention can be formulated.

## 2. Materials and Methods

### 2.1. Study Site and Sampling Points

**Figure 1:**
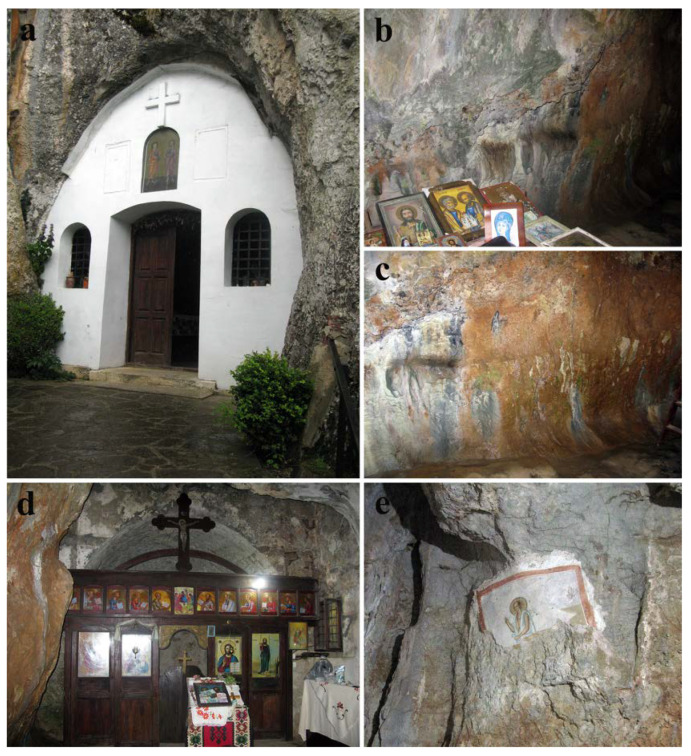
Cave Church of Sts. Peter and Paul: (**a**) exterior of the church; (**b,c**) southern cave walls; (**d**) wooden iconostasis; (**e**) fresco painting with a depiction of “The Bald-headed Jesus” on the northern wall.

The complexity of the geological composition of the territory of Republic of Serbia is reflected in numerous and diverse caves and caverns, many of which are protected due to their scientific and cultural relevance and importance [19]. These vary from large caves of several hundred meters deep to small sites, around ten meters deep, or even simple rock shelters. Stara planina, a complex mountain both in geological and tectonic context, is known for its numerous sinkholes and uvalas, as well as for the explored caves of small size. One such limestone cave (approx. 4 m W × 8 m D × 4 m H) repurposed as the church dedicated to saints Peter and Paul in XIII century, is located in the village of Rsovci (43°10′32″ N 22°46′35″ E), in the rocky massif of the Kalika hill, 22 km from the town of Pirot. It is part of “Stara planina” nature park, a category I protected area of international, national, or exceptional importance. Since 1981, the cave church has been classified as an immovable cultural property, under the direction of the Institute for Protection of Cultural Monuments of Niš, and represents a unique example of sacral architecture of this type in the eastern part of Serbia. The entrance to the cave was closed, at the beginning of the XXI century, in the form of a wall with doors and windows, while the altar niche was added on the eastern inner side (Figure 1a,d). The entrance wall is made of stone with minor interventions in concrete, while the floor is partially polished using cement (concrete). A part of a fresco painting on the north side has been preserved, depicting Christ in a light mandorla. He has a youthful face, a halo around his head, a white cloak accented with shades of blue, and is in a state of blessing (Figure 1e). The preserved fresco was most likely part of the “Vision of Peter of Alexandria” scene. Due to the atypical representation with a little hair on the side and a high forehead, this representation of Christ is known to the people as “The Bald-headed Jesus”. According to stylistic features, this fresco painting dates back to the post-Byzantine period [20]. Detailed insight into the cave church interior and immediate surroundings of the cave can be seen via the link: https://www.srbija3d.rs/lokacija_4/index.html, accessed on 22 October 2022.

For analysis purposes, samples were divided into samples taken from the fresco and around the fresco. The four sampling points on a fresco painting distinguished by the presence of different mineral pigments or showing evident symptoms of deterioration were: (depiction of Jesus (sample 01); entire fresco except depiction of Jesus (sample 02); gray discoloration on fresco (sample 03); fresco surface damage (samples 04-05)), while six samples around the fresco included—one sampling point on the wooden iconostasis with symptoms of fungal infestation in form of pinkish gray powdery surface [iconostasis (sample 06)) and five sampling points located on interior cave walls characterized by the presence of differently colored patinas and formed biofilm (blue-green deposits on stone wall under the fresco (sample 07); green patina on stone wall (sample 08); black deposits on stone wall (sample 09); white deposits on stone wall (sample 10); and pink deposits on stone wall (sample 11)).

### 2.2. Indoor Microclimate and Moisture Content Measurements

The temperature (T °C) and relative humidity (RH %) of the cave church interior were measured every 30 min during the 8 months period (from October 2020 to May 2021). For this purpose, testo 176P1 data logger was installed on the southern stone wall at a height of 250 cm from the floor, and probe was fixed to the surface of the stone.

Moisture content (%) of cave stone walls, fresco, and wooden iconostasis surfaces was measured in October 2020 using testo 606-2 measuring instrument set to limestone and wood mode, respectively.

### 2.3. Determination of Surface Contamination

To in situ assess the degree of total contamination of deteriorated stone walls, fresco and iconostasis, with microorganisms and organic residues, rapid ATP bioluminescence method was used [21]. For this purpose, ATP swabs and Lightning MVP portable luminometer (BioControl Systems) were used on all sampling points. Results were compared to the manufacturer’s provided reference scale and placed in one of the three categories of contamination (zone of cleanliness): clean zone (0.0–2.5); caution zone (2.5–3.0); and danger zone (3.0–7.5).

### 2.4. In Situ Microscopy

Direct observation of microbial growth and structural micro-impairments of stone wall, fresco and iconostasis surfaces was performed using a portable Dino-Lite Edge digital microscope AM7915MZTL. Image processing and measurements were achieved via DinoCapture 2.0 v1.5.39.A software.

### 2.5. Optical Microscopy

To collect samples for optical microscopy a non-aggressive adhesive tape method was applied [22]. Prior to microscopy, samples were stained with several drops of Lactophenol Cotton Blue and analyzed with a Zeiss Axio Imager M1 microscope using AxioVision Release 4.6 software.

### 2.6. Scanning Electron Microscopy (SEM)

For SEM, fragments of analyzed substrata were sampled via adhesive carbon tape on aluminum cylinders. Images were obtained at the University of Belgrade—Faculty of Mining and Geology using a JEOL JSM–6610LV microscope. Samples were gold coated (d = 15 nm, ρ = 19.2 g/cm^3^) with a Leica EM SCD005 sputter coater. Secondary electron and backscattered electron images were obtained using a W-filament gun, at 20 kV acceleration voltage in high-vacuum mode (15–30 μPa of pressure in the sample chamber) and magnifications ranging from 150 to 30,000×.

### 2.7. Isolation and Identification of Culturable Fungi

Samples for the isolation of culturable mycobiome were collected with sterile swabs. After sampling, swab tips were immersed in 2 mL microtubes (Sarstedt Inc., Newton, NC, USA) containing 1 mL of 30% glycerol Mueller–Hinton broth medium, frozen, and transported to the laboratory under sterile conditions. From each microtube, after 1 min treatment in a vortex mixer, 100 μL was pipetted to malt extract agar (MEA), oatmeal agar (OA), and potato dextrose agar (PDA), supplemented with streptomycin (500 mg L^−1^; streptomycin sulfate salt, Sigma-Aldrich) to suppress bacterial growth. In order to minimize sampling errors and to increase the likelihood of obtaining the highest possible diversity of culturable mycobiota, inoculation was carried out in triplicates. Inoculated media were incubated in a thermostat (UE 500, Memmert) at 25 ± 2 °C for 7 days. After the incubation period, morphologically different colonies were reinoculated on MEA and incubated under the same conditions to obtain axenic cultures. Obtained isolates were initially identified based on colony morphology and the microscopic characteristics of reproductive structures observed with the optical microscope Zeiss Axio Imager M1 using AxioVision Release 4.6 software. Identification of fungi was performed using the identification keys [23,24,25]. 

For molecular characterization, 50–100 mg of dry marginal mycelia were collected and used for DNA extraction according to the manufacturer’s instructions for a DNA Mini Kit (Qiagen, Hilden, Germany). PCR amplification of the *ITS* region was conducted using the primers ITS1/ITS4 [26], and Bt2a/Bt2b primers [27] were used for amplification of the *BenA* gene (only for *Aspergillus*, *Penicillium,* and *Fusarium* species). PCR amplification of *ITS* and *BenA* was performed as described in Savković et al. [28]. The resulting PCR products were separated by electrophoresis and purified (Purification Kit, Qiagen) for sequencing (Eurofins Genomics, Vienna, Austria). Sequences were compared with other related sequences from the National Center for Biotechnology Information (NCBI) database using the BLASTn program. Phylogenetic analysis was conducted using the ClustalW algorithm integrated with MEGAX software and the tree was constructed using maximum likelihood joining phylogeny. The reliability of the constructed phylogenetic tree was evaluated with 1000 replications for branch stability.

### 2.8. Biodegradation Plate Assays

To assess biodeterioration potential in vitro, 30 fungal isolates were screened using qualitative assays. All isolates were screened for carbonate dissolution on CaCO_3_ enriched agar plates, according to Albertano and Urzì [29], while production of acidic metabolites was completed on creatine sucrose agar (CREA) and minimal Czapek broth (mCzB), as described in Savković et al. [30] and Borrego et al. [31]. Extracellular pigment production was evaluated on OA [30] and hue was determined per ISCC-NBS color palette (https://www.w3schools.com, accessed on 15 January 2022). Isolates from fresco were evaluated for proteolytic activity using casein agar (CN) medium which was prepared by the protocol given in Saran et al. [32] and Pangallo et al. [33]. Isolates from wooden iconostasis were screened for cellulolytic and hemicellulolytic activity on media enriched with carboxymethyl cellulose sodium salt or xylan from beech wood, respectively, as proposed by Teather and Wood [34] and Liang et al. [35]. Additionally, isolates were evaluated for ligninolytic activity, which was performed on PDA containing ABTS and CuSO_4_ to detect laccase activity, Mannitol agar medium (MAM) supplemented with azure B to detect lignin peroxidase activity, and Czapek agar (CzA) containing phenol red, MnCl_2_, and H_2_O_2_ for detection of Mn-dependent peroxidase activity [36,37,38].

### 2.9. Amplicon Sequencing

#### 2.9.1. DNA Extraction, Library Preparation, and NGS Sequencing

Special sterile swabs were taken from all of the sampling points and protected by DNA/RNA shields (Zymo Research, Irvine, CA, USA) during transport. The extraction of ultra-pure DNA was completed using the Zymo BIOMICS DNA Mini Kit (Zymo Research) for several swabs from each sampling point, following the manufacturer’s protocol. The DNA yield was measured using Qubit Fluorometric Quantitation (Qubit 4 Fluorometer, Invitrogen™, Waltham, MA, USA). Library preparation, using Nextera XT Index Kit (FC-131-1096), and amplicon sequencing step was performed using a 2×300 bp paired-end run on a MiSeq Sequencer, according to manufacturer’s instructions (Illumina) in commercially available service (FISABIO, Valencia, Spain). To target the *ITS* II region, the primers [39] were as follows: forward primer ITS3_KYO2 (5′- GATGAAGAACGYAGYRAA-3′), and reverse primer ITS4_KYO1 (5′-TCCTCCGCTTWTTGWTWTGC-3′).

#### 2.9.2. Sequence Data Process and Taxonomy Annotation

Quality-based filtering/trimming was performed using DaDa2 R package [40]. Primer trimming was performed with BBduk [41] using literal primer sequences as follows: ktrim = l—left trim; hdist = 1—hamming distance of 1—allow one mismatch to primer sequence; copy undefined—clone the reference primer sequences to represent every possibility for the degenerate bases; mm = f—disable mask middle option which is turned on by default and ignores the middle base of a kmer; rcomp = f—do not search reverse complement; and k = 15—use k-mer length 15. All sequences having more than 3 for forward and 2 for reverse strand expected errors (calculated as sum(10^(−Q/10))—where Q is the quality score), were discarded (argument:maxEE = c(3, 2)), as well as sequences shorter than 50 bp. Sequence merger was performed with a minimum overlap of 20 bases without mismatches. After merger, chimeric sequences were removed using default parameters in the DaDa2 R package. All sequences with a length shorter than 280 bp and longer than 367 bp were removed. Contaminant sequences were identified using the R package decontam [42] using the “combined” method which combines the frequency and prevalence probabilities by using Fisher’s method to identify contaminants (Table S1).

ITS taxonomy assignment was performed using the UNITE database which includes global and 97% singletons (https://dx.doi.org/10.15156/BIO/786369, accessed on 20 December 2021). The RDP naive Bayesian classifier [43] with default options as implemented in DaDa2 package was used to classify taxonomy up to the genus level. In addition, amplicon sequence variants, with high abundance and ambiguous taxonomy assignment, were annotated based on the BLAST best hit in the NCBI nucleotide database up to the species level annotation. Main diversity data reported have been obtained by considering the whole dataset, including singletons and/or under-represented taxa.

#### 2.9.3. Statistical Analysis

Sequence diversity within samples (alpha diversity) was estimated using the phyloseq R package [44] at the amplicon sequence variant (ASV), genus, family, and phylum levels. Alpha diversity was shown through estimators Shannon and Gini–Simpson. Observed and estimated richness was determined according to the following estimators: number of observations (OBS), Chao1, and ACE. The Kruskal–Wallis test by ranks was used for testing significance among the samples obtained from fresco (samples 01-05) and from the cave interior including iconostasis (hereinafter: around fresco, samples 06-11). For visualization of beta diversity shared across sample communities, a double principal coordinate analysis—DPCoA [45] was used. This method requires phylogenetic distances of ASVs in addition to just abundances, and for the phylogenetic tree, ASVs were aligned using DECIPHER:AlignSeqs with default arguments [46]. Apart from this approach, a variance stabilizing transformation is implemented instead of rarefaction, and the multidimensional scaling transformation (MDS) was performed using Euclidean distances. For the visualization of the distances among samples, weighted UniFrac distances [47], which take into account phylogenetic distances between observed taxa in addition to taxa counts during the computation, were used. Node size corresponds to the number of connections that the node has, while the edge width corresponds to the distance among samples (closer distances equal wider edges). Nodes that were not connected to other nodes are not shown. For all analyses stated above, prior to computation of the diversities and distances, samples were rarefied to even depth (sample with the lowest number of reads—Figure S1).

The relationships among tax categories were visualized based on co-occurrence using package cooccur [48]. Only statistically significant co-occurrence patterns were shown. Prior to running the co-occurrence analysis all counts that were less than 5 were converted to 0 (categories with a count of less than 5 were considered absent). Additionally, a genus-level network based on weighted Jaccard distance (computed as 2B/(1 + B), where B is Bray–Curtis dissimilarity) was computed. Distances lower than 0.6 are shown as edges. Categories present in any sample with at least 0.5% were taken into account for both analyses.

Differential abundance analysis was performed using DESeq2 package [49]. Starting from the genus level aggregation the package metacoder [50] was used to generate an abundance matrix at all taxa levels. The matrix was filtered by removing taxa that did not have at least 5 reads in at least 3 samples. DESeq was performed using Wald-test statistic, log2-fold change threshold of 2, Benjamini and Holceberg [51] p-adjustment, while all other arguments were left at default. *p*-adjusted values < 0.05 were considered significant. Shrinkage of log2-fold changes was performed using the apeglm method [52].

## 3. Results

### 3.1. State of the Investigated Cave Church

**Figure 2:**
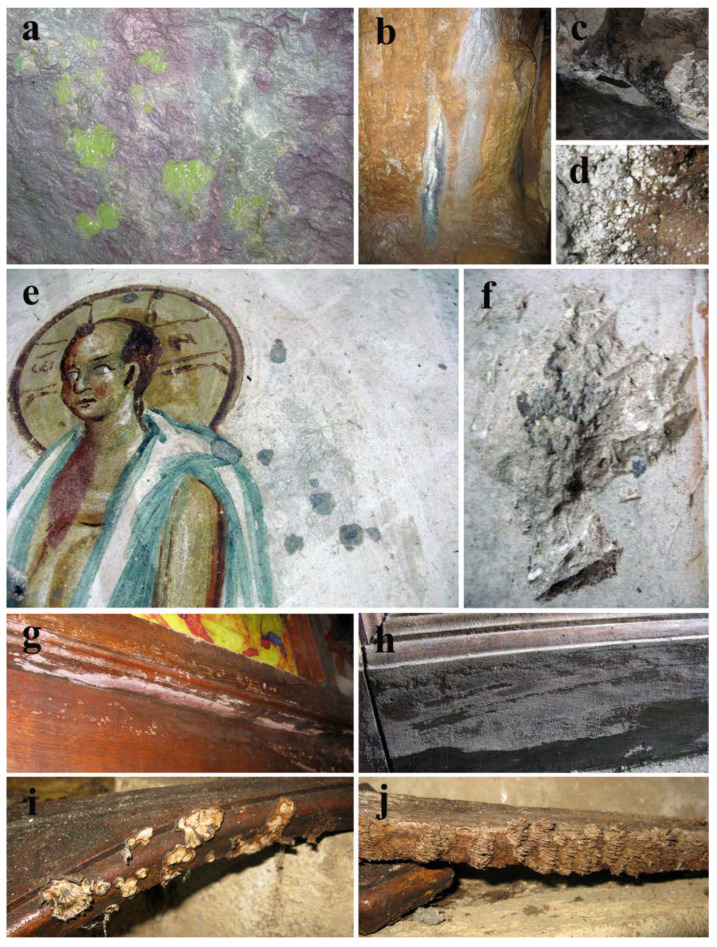
Alterations observed on the various substrata within the Cave Church of Sts. Peter and Paul: (**a**) green biofilm and pink patina on northern stone wall; (**b,c**) differently pigmented patinas; (**d**) surface salt efflorescence; (**e**) wet grayish surface discolorations; (**f**) loss of fresco painted layer and plaster; (**g,h**) fungal infestation on wooden iconostasis; (**i,j**) fruiting bodies of *Stereum gausapatum* and other wood-decay fungi on iconostasis fragments.

There is an evident difference between the northern and southern walls within the cave. While the southern wall is mostly dry and lacking apparent alterations of the stone substrata, the northern wall is quite moist, with known occasional leakage of water, and with variously colored patinas and biofilm developed on the surface (Figure 2a–c). In several areas, surface salt efflorescence was observed (Figure 2d). On the upper right side of the northern stone wall, at about 3 m height, a preserved part of the “The Bald-headed Jesus” fresco painting is present. Only the upper half of the fresco remains, with both the painted layer and plaster of the bottom half completely missing. On the painted surface, numerous fungal-induced wet grayish discolorations of variable size and form are present (Figure 2e). Substantial damage of the painted layer, characterized by the loss of the painted layer uncovering the plaster below, is present in the upper right side of the preserved fresco painting (Figure 2f). 

On the eastern side of the cave a wooden iconostasis, made of dark lacquered wood, is present separating the altar niche from the rest of the cave, for the most part unscathed. The bottom half of the iconostasis is, however, impaired and the presence of pinkish gray powdery mass covering substantial surfaces is clearly visible (Figure 2g,h). Furthermore, several wooden fragments are torn off from the iconostasis partition and covered with fully developed, albeit dry, fruiting bodies of *Stereum gausapatum* and other wood-decay fungi (Figure 2i,j).

### 3.2. Indoor Church Microclimate and Material Surface Moisture Content

**Figure 3:**
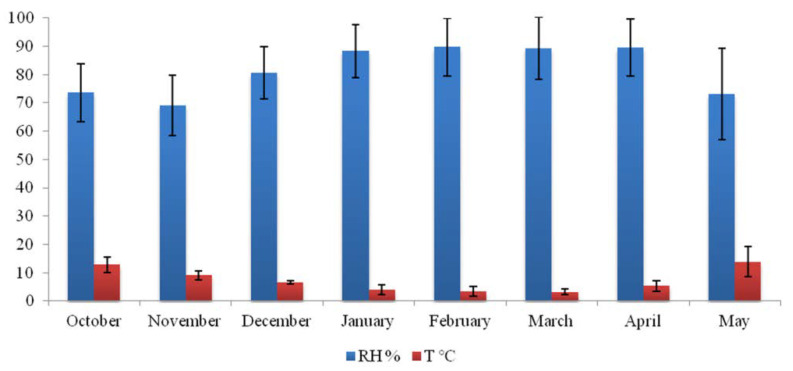
Relative humidity (RH%) and temperature (°C) fluctuations within Cave Church of Sts. Peter and Paul.

Based on 10,651 measured values during the 8 months period, documented T was in the range of 0.2 to 38.2 °C, with an average temperature of 6.7 °C; while RH was ranging from 26.6 to 99.9% (average of 82.4%) (Figure 3).

Measured values of surface moisture content for limestone cave walls, fresco, and wooden iconostasis are summarized in Table 1. Surface moisture content values were in the range of 20.1 to 23.8% and 1.6 to 3.0% for cave walls and frescos, respectively. The value of 17.1% was documented for wooden iconostasis.

### 3.3. Surface Microbial Contamination

Zone of cleanliness values, as indicators of the degree of total contamination of stone walls, fresco, and iconostasis, with microorganisms and organic residues, were in the range of 5.3 to 6.4 (Table 1). All documented values were according to the manufacturer’s provided reference scale placed in the “Danger zone” (3.0–7.5) category of contamination. Iconostasis surface was the most contaminated (6.4) as was expected due to visible fungal proliferation on the surface, followed by a stone wall (avg. 5.6) and fresco (avg. 5.4) surfaces.

### 3.4. In Situ Assessment of Micro-Impairments and Surface Microbial Growth

**Figure 4:**
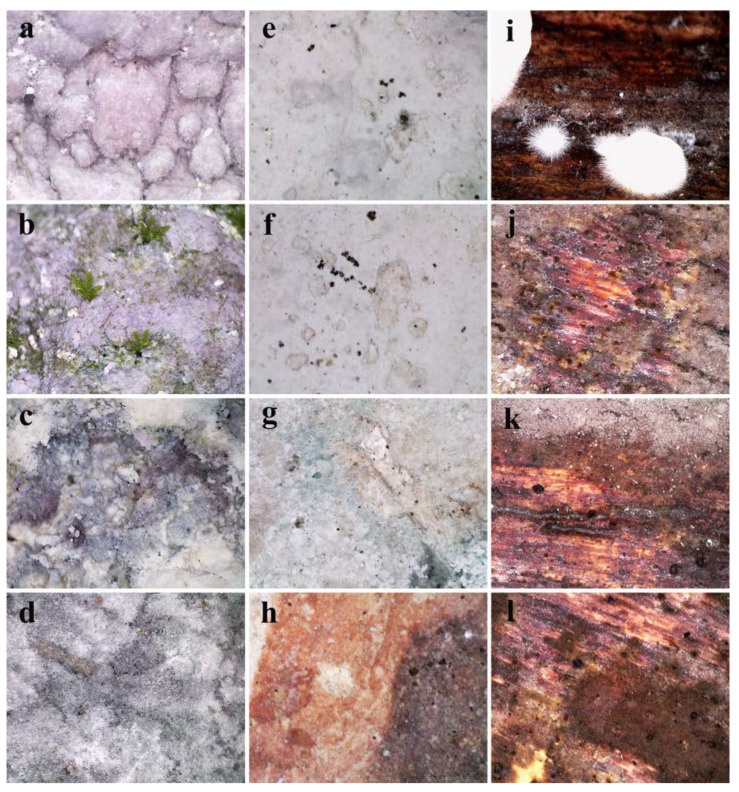
In situ microscopy of deteriorated surfaces in the ambient of Cave Church of Sts. Peter and Paul: (**a**–**d**) visible biofilm with salt efflorescence on stone walls; (**e**–**h**) biopitting symptoms on fresco painting surface; (**i**–**l**) pronounced fungal growth on wooden iconostasis.

Direct observation of the southern stone wall within the cave church, via portable in situ microscopes, revealed a rough surface covered with salt efflorescence resembling pink-hued snowballs (Figure 4a), with occasional moss gametophyte and visible thread-like protonema (Figure 4b). Furthermore, in general, observed stone surfaces highly deteriorated with visible structural impairments, i.e., variously colored granulated surfaces interspersed with conspicuous filamentous salts (Figure 4c,d). Predominant deterioration symptoms, observed on the surface of the investigated fresco, were loss of fragments of the painted layer along with clear signs of biopitting occasionally filled with melanized fungal structures of typical meristematic clusters characteristic of microcolonial (rock-inhabiting) fungi (Figure 4e–h). The surface of the wooden iconostasis was characterized by the presence of immature basidiomycetes fruiting bodies (Figure 4i) and was extensively covered with a pinkish-gray fungal mat with apparently present stromata with visible dark perithecia (Figure 4j–l).

### 3.5. Fungal Proliferation on Fresco and Iconostasis Surfaces

**Figure 5:**
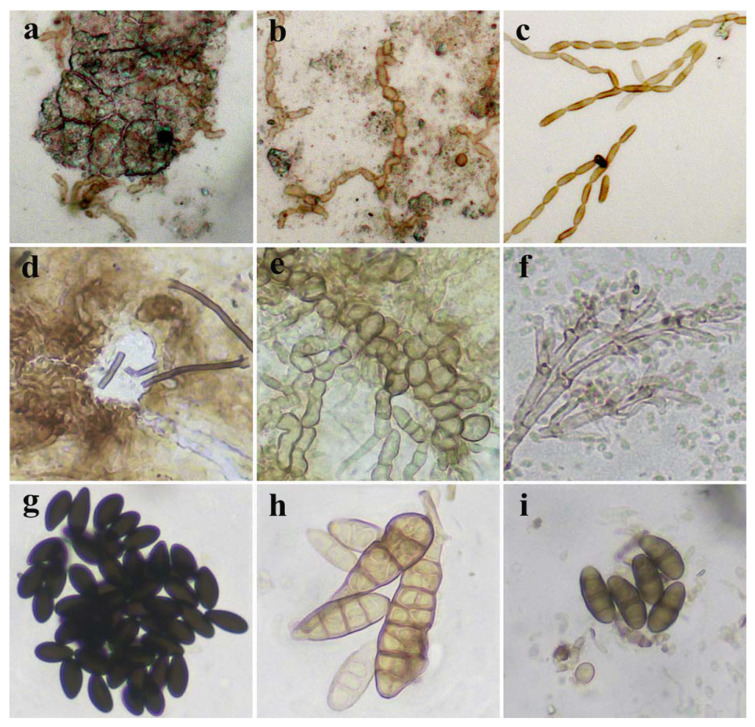
Optical micrographs of fungal structures in adhesive tape samples from fresco painting (**a**–**c**) and wooden iconostasis (**d**–**i**): (**a**–**c**) *Neodevriesia* conidial chains on deteriorated fresco surface; (**d**,**e**) fungal structures permeating lacquered wood surface; (**f**) branched geniculate terminal conidiophore of the anamorphic state of *Hypoxylon*; (**g**) inequilaterally ellipsoid *Hypoxylon* ascospores in mass; (**h**) Alternaria-like dictiospores; (**i**) Dematiaceous hyphomycetes fragmospores, (400×).

Adhesive tape samples, taken from wet grayish discolorations of the fresco-painted layer (Figure 1e and Figure 2e), have revealed numerous melanized Cladosporium-like conidial chains in the interspace between painted layer and the plaster below, possibly belonging to the fungus of *Neodevriesia* genus (Figure 5a,b). Furthermore, branched, fragile conidial chains, composed of golden-brown conidia with or without visible dark septum, are also observed proliferating on the surface of the painted layer (Figure 5c). On the other hand, a mass of melanized, fragmented hyphae with chlamydospores was observed permeating below the lacquered wood surface (Figure 5d,e). Optical microscopy of adhesive tape samples taken from a fungal mat resembling pinkish gray powdery surface (Figure 2g,h), a dominant deterioration symptom present on the wooden iconostasis, has shown a mass of branched, pale grayish-brown geniculate terminal conidiophores with numerous pale grayish-green conidia of the anamorphic state of *Hypoxylon* (Figure 5f) together with black-brown inequilaterally ellipsoid *Hypoxylon* ascospores (Figure 5f). The presence of Alternaria-like dictiospores and melanized ascospores of *Pleosporales* was also observed (Figure 5h,i). 

**Figure 6:**
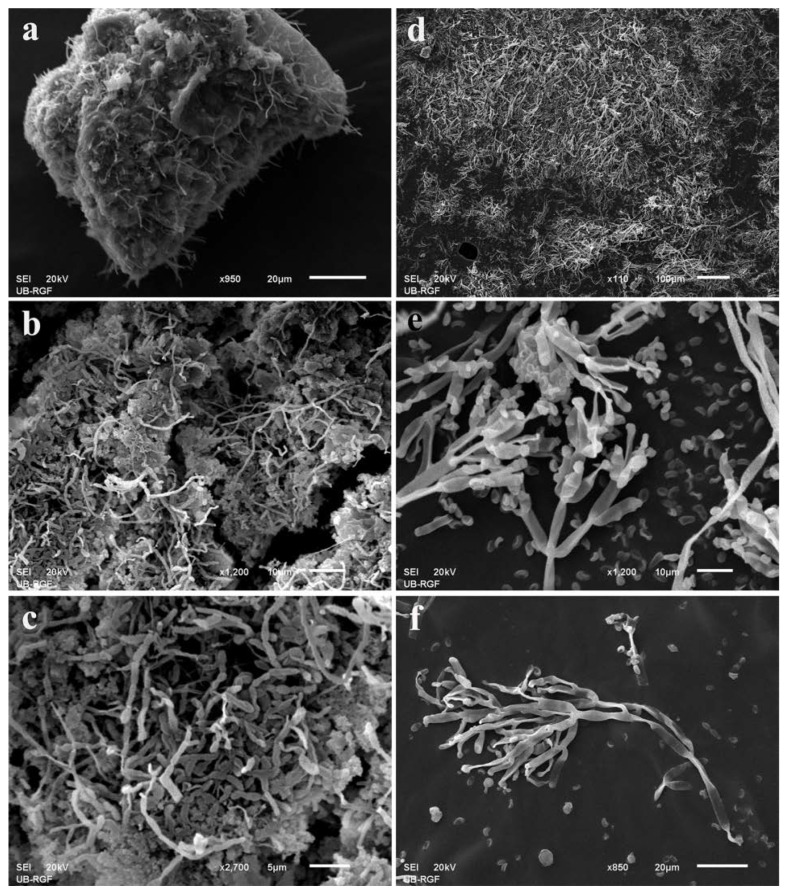
Scanning electron micrographs of fragments from deteriorated fresco painting and wooden iconostasis: (**a**–**c**) *Neodevriesia* conidial chains from wet grayish fresco discoloration; (**d**–**f**) branched conidiophores with conidia of the anamorphic state of *Hypoxylon*.

Observations made with optical microscopy were additionally confirmed with SEM: *Neodevriesia* conidial chains were found permeating the plaster below the painted layer (Figure 6a–c), while on the iconostasis a mass of branched conidiophores of the anamorphic state of *Hypoxylon* (Figure 6d) and details of conidiogenous apparatus (Figure 6e,f) were observed.

### 3.6. Culturable Mycobiome

**Figure 7:**
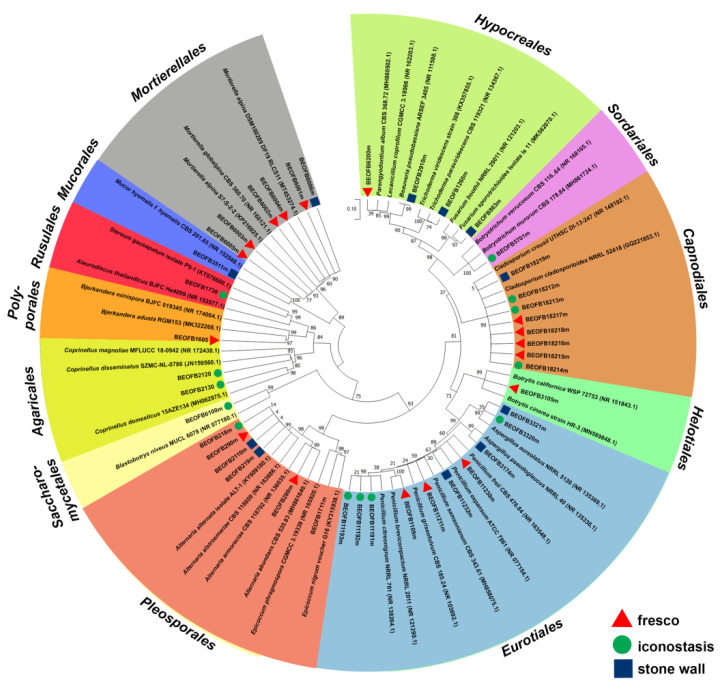
Maximum likelihood tree of *ITS* region of culturable fungi isolated from the Cave Church of Sts. Peter and Paul.

A total of 24 fungi from 17 genera were determined as part of the culturable mycobiome of different substrata within the cave church (Figure 7, Table S2). The greatest diversity was documented on the stone walls (12 species), followed by fresco (10) and iconostasis (8). The majority of isolated fungi were *Ascomycota* (73.81%), while only 16.67% and 9.52% belonged to phyla *Zygomycota* and *Basidiomycota*, respectively. With five documented species, the genus *Penicillium* was the most diverse, while isolates of *Cladosporium* and *Penicillium* genera were the most frequently isolated.

Phylogenetic analyses showed that all *Ascomycota* members clustered together (bootstrap value (bv) = 75, Figure 7). *Aspergillus* and *Penicillium* species formed a well-supported clade (bv = 99) as members of the order *Eurotiales*. Likewise, *Trichoderma harzianum*, *Fusarium sporotrichioides*, *Beauveria pseudobassiana,* and *Parengyodontium album* clustered together as the members of *Hypocreales* clade (bv = 87). *Cladosporium* species clustered in a well-supported *Capnodiales* clade (bv = 99), as well as the members of *Alternaria* and *Epicoccum* species (*Pleosporales* clade, bv = 99). Among *Basidiomycota*, *Coprinellus* species clustered together with *Bjerkandera* and *Stereum* species in a well-supported *Basidiomycota* clade (bv = 84). Finally, *Mortierella alpina* isolates clustered with *Mucor hyemalis* in *Zygomycota* (bv = 89).

### 3.7. Biodegradative Profile of Isolated Fungi

Among 30 tested isolates, 16 (53.33%) demonstrated at least 1 positive reaction in biodegradation assays (Table 2). Acid production was observed in eight isolates (26.67%) with a reduction in pH of the liquid medium from 4.27 to 6.73, while carbonate dissolution was documented only for *Penicillium samsonianum* BEOFB11211m and *Cladosporium cladosporioides* BEOFB18216m, isolated form fresco surface. Likewise, coupled cellulolytic and hemicellulolytic activity is observed in *Alternaria alternata* BEOFB218m, *Botryotrichum murorum* BEOFB5701m, and *Stereum gausapatum* BEOFB1730 isolated from iconostasis, while *Coprinellus disseminatus* BEOFB2120 had only hemicellulolytic activity. Ligninolytic activity is observed in six fungal isolates from iconostasis (20.00%). Proteolytic activity was documented for five isolates (16.67%), while pigment production was demonstrated for six (20.00%). The colors of produced pigments were mostly in different hues of yellow: deep greenish yellow (#9b9400) in *Aspergillus aureolatus* BEOFB3320m and BEOFB3321m, vivid yellow–green (#8db600) in *Penicillium freii* BEOFB11230m, brilliant greenish yellow (#e9e450) in *Beauveria pseudobassiana* BEOFB2910m, strong orange yellow (#eaa221) in *S. gausapatum* BEOFB1730 and strong greenish yellow (#beb72e) in *Bjerkandera adusta* BEOFB1605.

### 3.8. Diversity of Microbial Communities

A total of 1,505,219 raw sequences were obtained from the ITS libraries sequencing (from 106,494 to 222,761 per sample). After denoising, quality filtering, length trims, and sequence decontamination, the number of reads ranged from 76,734 up to 190,144 (Table S1). The mean length of obtained ASV sequences was 323. Based on alpha rarefaction curves (Figure S1), since there were evident differences in reads among samples, diversity indices were estimated after rarefaction to even depth according to the sample with the lowest number of reads (sample 07). Alpha diversity indices were calculated for OBS, Chao1, ACE, Shannon, and Gini–Simpson at phylum, family, genus, and ASV levels (Table 3). The diversity indices on average, Shannon and Gini–Simpson, indicated lower diversity in samples taken from the fresco itself, while richness estimators (Chao1 and ACE) reflected the higher fungal richness (at least double) in the samples taken around the fresco, especially at the family, genus, and AVS taxa levels. However, statistically significant differences, for the Shannon (*p* = 0.02) and Simpson (*p* = 0.02) indices of the fresco and around it, according to the Kruskal–Wallis test were observed only at the phylum level (*p* < 0.05). Reviewing the samples individually, it was noticed the highest fungal richness at the family, genus, and AVS taxa levels for the 04-05 sample taken from the fresco surface damage, while the same is obtained for the samples from the cave interior wall deposits in increasing order 11 > 10 > 07. Sample 03 collected from the gray discoloration on the fresco itself, showed the lowest richness and diversity according to the Shannon index, and a similar is scored for sample 06 from the iconostasis (Table S3). Sample 08 obtained from the green patina showed, among all samples from the wall deposit, the lowest richness and diversity.

**Figure 8:**
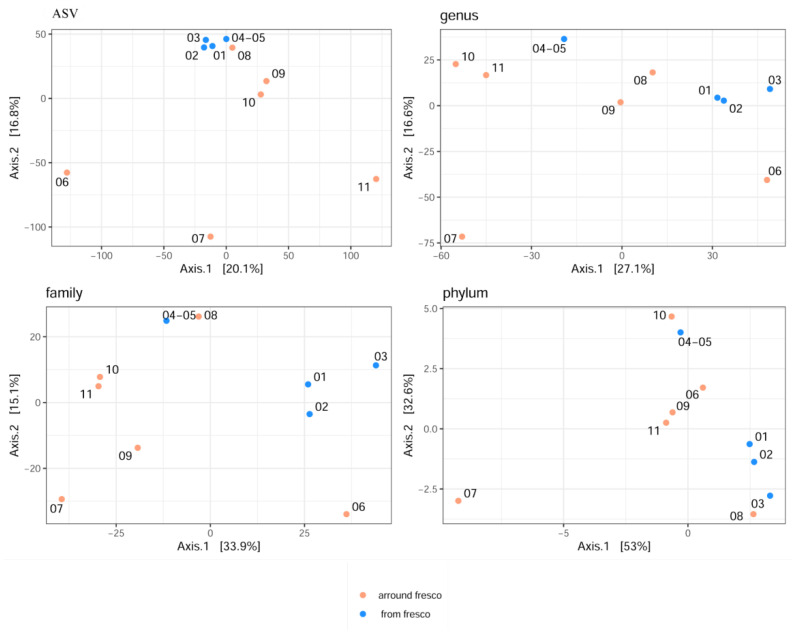
The MDS (PCoA) plot created with Euclidean distances at the phylum, family, genus, and ASV taxa levels, showing the variability between samples.

For visualization of beta diversity, the initial analysis was performed with Bray–Curtis dissimilarities and samples were rarefied to even depth (variability between samples is shown with the DPCoA plot in Figure S2). Based on the results, it can be concluded that the samples from the fresco environment are jointly together, as well as the samples obtained from the side walls of the church in terms of microbial diversity at all taxonomic levels. The furthermost distance was achieved for samples 06 and 08, especially at the family, genus, and ASV taxa levels. Moreover, samples 09 and 11 were closely related to the samples from the fresco. Additionally, appropriate variance stabilization was performed later, and the results were presented by performing MDS (PCoA) analysis based on Euclidean distances (Figure 8).

From the PCoA plot, it can be observed that fresco’s samples 01, 02, and 03 were closely related to each other according to PCoA1 at phylum, family, and genus taxa levels (explaining 53%, 33.9%, and 27.4% of variability, respectively). Samples 06 and 11 were identified as outliers and more dissimilar to other samples, according to both axes at the ASV level, including sample 07 based on the second axis, as well.

**Figure 9:**
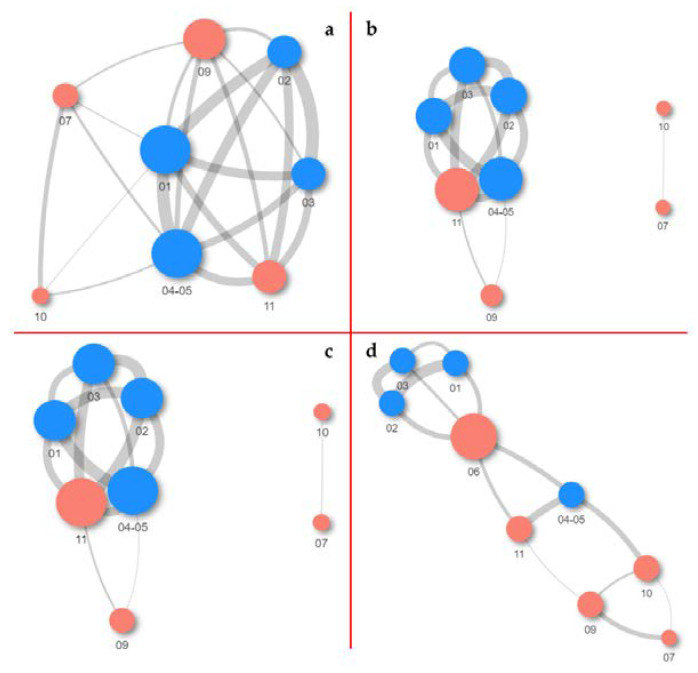
Network analysis of samples with weighted UniFrac distances at the: (**a**) ASV; (**b**) genus; (**c**) family; and (**d**) phylum taxa levels. Node size corresponds to the number of connections, while edge width corresponds to the distance among samples (closer distances equal wider edges). Red nodes are from the samples around the fresco, while blue nodes are the fresco’s samples.

Weighted UniFrac distances were used to visualize the distances among samples (Figure 9). Based on the results obtained at the family and genus taxa levels, samples 07 and 10 were quite far from the others and had a single connection but were sufficiently distant among themselves (proximity value of 0.2). Samples 01 and 04-05 at the ASV level had the highest number of connections with the other samples (the number of connections was 7) and were closest among themselves, according to the proximity value (0.061). The same proximity was noted for samples 02 and 03; 02 had the longest distance from samples 04-05 with 0.098; likewise, sample 03 from sample 01 with a 0.117 distance value. Additionally, samples 09 and 11 clustered together with the samples from the fresco, supporting the results of DPCoA analysis, among themselves were quite distant (0.212). At the genus and family taxa levels, the situation was similar for samples 04-05 and 11, along with five connections with the rest samples, and the last one (11) had a closer distance to the fresco’s samples. Sample 6, although somewhat distant, achieved five connections with samples 01, 02, 03, 04-05, and 11 only at the phylum level; in all other taxa comparisons, it was excluded from the analysis due to its excessive distance from the others.

### 3.9. Total Microbial Composition

The phylum *Ascomycota* dominated all samples (79.9–99.7%), while *Basidiomycota* was in higher abundance on the cave walls around the fresco. The phylum, which does not belong to fungi, *Chlorophyta* (with the algal species *Pseudostichococcus monallantoides*, according to BLAST results) was predominant for green patina on the stone wall (sample 08). 

**Figure 10:**
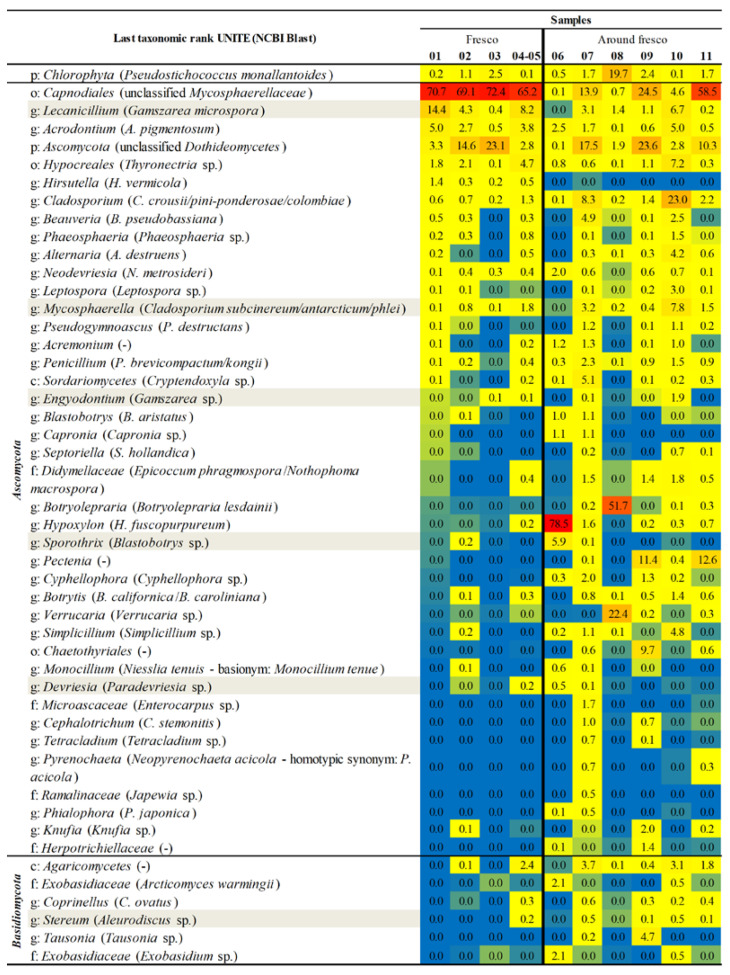
Relative abundance of fungal taxa at the last taxonomic rank according to UNITE database and NCBI assignments at the species level. Relative abundances of fungal taxa are present at all proportion levels, and different colors indicate low or high abundance. (p)—phylum; (c)—class; (o)—order; (f)—family; (g)—genus; (-)—not determined. The gray color indicates differences between UNITE and NCBI results. Only taxa with a total percentage abundance above 0.5% for all samples were included. Blue, yellow, and red colors—lowest, medium, and highest RA.

The fungal taxa that dominate the fresco’s samples communities in higher or moderate relative abundance (RA) are order *Capnodiales* with unclassified *Mycosphaerellaceae* family, unclassified class *Dothideomycetes* (more characteristic for the entire fresco except for depiction of Jesus and gray discoloration on fresco—samples 02 and 03), as well as taxa *Lecanicillium, Acrodontium,* and *Hirsutella* (relatively higher abundant in samples 01, 02 and 04-05; the highest in a sample from the depiction of Jesus) which according to NCBI Blast results could be associated with *Gamszarea microspora, A. pigmentosum,* and *H. vermicola,* respectively (Figure 10).

According to NCBI alignment of the sequence obtained for the order *Capnodiales* (which was the most abundant across all fresco samples), it was closest to the *Mycosphaerellaceae* and *Teratosphaeriaceae* families, i.e., the genera *Annellosympodiella*, *Neodevriesia*, and *Catenulostroma*. Additionally, order *Hypocreales* (NCBI-*Thyronectria* sp.) was characteristic for mentioned samples as well. Slightly more abundant in fresco surface damage (04-05) than in other fresco samples were *Cladosporium*, *Phaeosphaeria*, *Alternaria*, *Mycosphaerella*, *Penicillium*, and unclassified class *Agaricomycetes*. Contrary to that, in a sample taken from the iconostasis (06), we observed dominance of the genera *Hypoxylon* (*H. fuscopurpureum*), *Sporothrix* (*Blastobotrys* sp.), *Acrodontium* (*A. pigmentosum*), and unclassified family *Exobasidiaceae* (per NCBI could potentially annotate as *Arcticomyces warmingii* and *Exobasidium* sp.). It is interesting to note that the sample taken from gray discoloration on fresco (03) was relatively poorly abundant of detected taxa such as *Beauveria* (*B. pseudobassiana*), *Phaeosphaeria* (*Phaeosphaeria* sp.), and *Alternaria* (*A. destruens*) among many others.

The opposite situation occurred for the samples from the points located on interior cave walls. As long as the taxa *Capnodiales* with unclassified *Mycosphaerellaceae* family and unclassified class *Dothideomycetes* were ubiquitous for the samples 07, 09, and 11 (potentially indicating the origin of fresco’ colonization), other taxa such as *Cladosporium* (*C. crousii*/*pini-ponderosae*/*colombiae*), *Beauveria* (*B. pseudobassiana*), *Mycosphaerella* (*Cladosporium subcinereum*/*antarcticum*/*phlei*), *Penicillium* (*P. brevicompactum*/*kongii*), and class *Agaricomycetes* were attached for samples 07 and 10 with higher RA. Additionally, sample 07 was characterized with higher RA with class *Sordariomycetes* (*Cryptendoxyla* sp.), and genus *Cyphellophora*. For samples 09 and 11, genus *Pectenia* in higher abundance simultaneously co-occurred, while order *Chaetothyriales*, unclassified family *Herpotrichiellaceae* and genus *Tausonia* were present in higher abundance only in sample 09. Higher dominance of *Chlorophyta* (*P. monallantoides*—phycobiont of lichenized fungus *Verrucaria* sp.), and lichenized fungi *Botryolepraria* (*Botryolepraria lesdainii*), and *Verrucaria* (*Verrucaria* sp.) were noted only for sample 08 taken from an area of green patina. The most abundant taxa related to the composition within sample 10 were also *Lecanicillium*, *Acrodontium*, *Alternaria*, *Simplicillium*, and order *Hypocreales*.

### 3.10. Co-Occurrence and Relative Abundance Analyses

**Figure 11:**
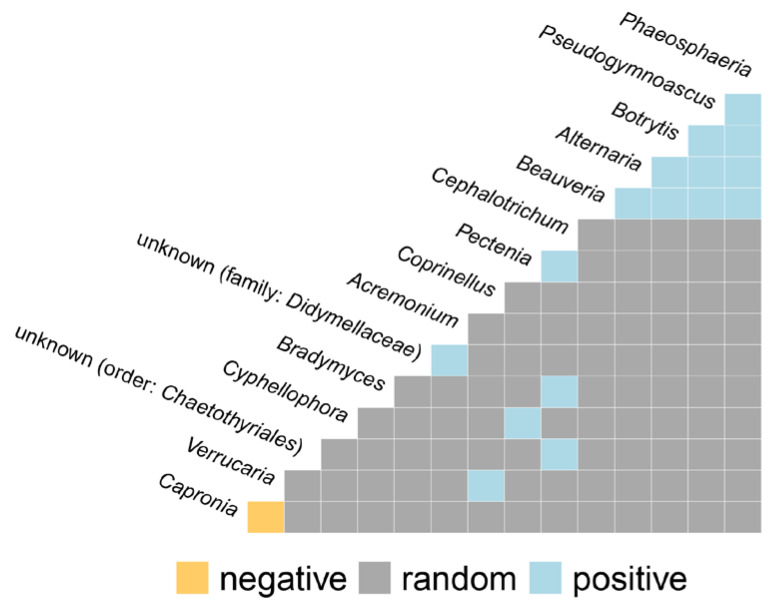
Co-occurrence analysis between detected taxa with positive, negative, and random interactions shown. Only those taxa that were present in any sample with at least 0.5% of occurrence were analyzed.

**Figure 12:**
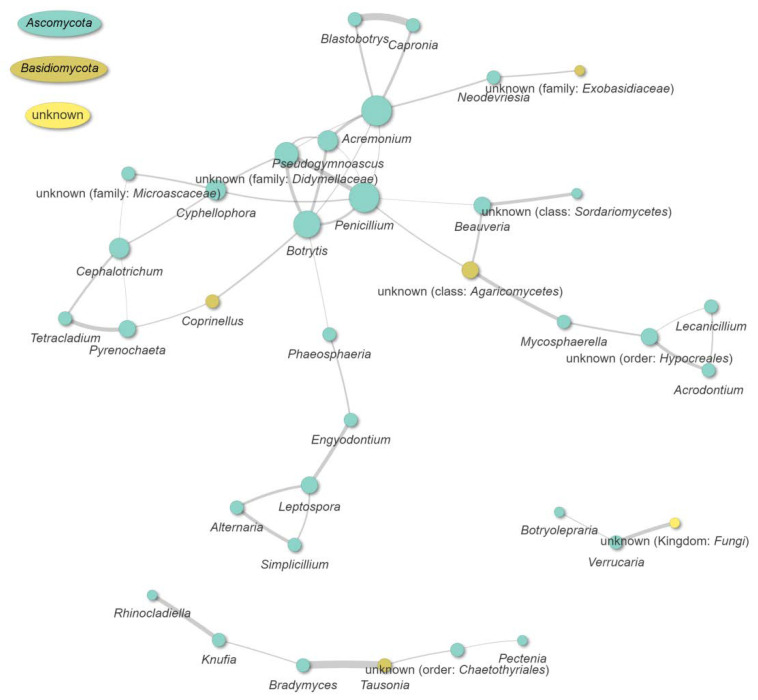
A genus-level network based on weighted Jaccard distance. Distances lower than 0.6 are shown as edges. Only those taxa that were present in any sample with at least 0.5% of occurrence were analyzed.

Relationships between taxonomic categories (positive, negative, and random interactions) were visualized based on the co-occurrence of taxa (“co-occurrence” analysis) as shown in Figure 11. Based on the statistically significant co-occurrence patterns, a positive correlation was observed among the genera *Beauveria*, *Alternaria*, *Botrytis*, *Pseudogymnoascus*, and *Phaeosphaeria* (with four positive connections for each of them). A positive correlation was also observed between *Cephalotrichum* (three connections) with *Pectenia*, *Bradymyces*, and an unknown taxon from the order *Chaetothyriales*. A negative correlation was observed only between the genera *Verrucaria* and *Capronia*. However, *Verrucaria* also has a simultaneous positive interaction with basidiomycetous *Coprinellus*. Additionally, a genus-level network based on weighted Jaccard distance computed on Bray–Curtis dissimilarity indicated numerous positive connections among different taxa (Figure 12). As can be noticed, the highest number of connections were achieved for *Penicillium* and *Acremonium* (each with seven positive connections), while the unknown taxa from the family *Didymellaceae* were tightly connected to *Penicillium* and *Botrytis*. Similar tight bonds are shown between *Blastobotrys* and *Capronia*, *Bradymyces* and *Tausonia*, as well as *Tetracladium* and *Pyrenochaeta*. *Leptospora* formed three positive connections with *Alternaria*, *Simplicillium*, and *Parengyodontium*, while unknown taxa from the order *Hypocreales* were positively connected with *Acrodontium*, *Lecanicillium*, and *Mycosphaerella*. *Verrucaria* has shown a slightly distant positive correlation with *Botryolepraria*, while *Neodevriesia* was closest to *Acremonium*. 

Differential abundance analysis was used to generate a matrix of RA of statistically significant species at all taxonomic levels according to sample origin (Table 4). It was established that three known genera *Pyrenochaeta*, *Phialophora*, and *Rhinocladiella* were significantly present only in samples around the fresco, while only *Hirsutella* was exclusively important for the fresco environment itself and mightily be defined as “core” species. 

## 4. Discussion

During the eight-month measuring period, the indoor temperature of the cave church was in the range of 0.2 to 38.2 °C, which is problematic having in mind the presence of fresco painting and the recommended range of temperatures for the preservation of frescoes which is from 6 to 25 °C [53]. On a daily basis, the indoor temperature did not change significantly and followed the seasonal trend of changes in outdoor temperature. This was expected since the church is part of the rock mass closed by a wall and therefore has high thermal inertia. The daily variation in the temperature usually did not exceed 1.5 °C; however, a frequent increase in daily temperature variation was noticeable after the middle of January and during the upcoming season, which correlates with the more frequent use and entry of visitors. Due to frequent liturgies during winter, it is sporadically heated to enable the comfortable presence of believers. Such sudden daily temperature variations, above recommended daily variation of 1.5 °C, contributed to the degradation of the material and originate from the human factor, and as such can be eliminated by corrective measures. Furthermore, during February and March, the temperature inside the church was constantly below 6 °C, sometimes reaching values close to 0 °C, creating a permanent danger of condensation processes and freezing of water present in the fresco plaster. This factor cannot be influenced by passive measures and was one of the main reasons for the substantial loss of fresco in the past. On the other hand, the documented minimum RH value was 26.6% and refers to the moment when the indoors of the church is extremely heated, while the recorded maximum of 99.9% is often repeated at longer intervals and for several days in January, February, March, and April. The recommended RH values for preserving fresco paintings are in the range of 45 to 60% [53]. RH values sometimes dropped to 56% and correspond to warmer periods and the daily use of the church. Although the RH values are mostly outside the recommended range, the daily variations are stable and do not change over 5% if there is no activity within the church. Most of the measurement intervals of the year RH values are above 70%, very often with long-term maximum saturation of water vapor, which leads to the appearance of moisture condensation in the fresco. The maximum values indicate the probable inflow of water into the walls by draining atmospheric water through porous limestone rocks. Aside from the issue of T and RH variation being physical agents of deterioration, for the most part of the measuring period both T and RH were in the range that favors fungal growth [54]. This growth is also certainly facilitated by the highly documented values of surface moisture content in cave walls and iconostasis. In the case of limestone walls, it is most likely due to drainage of water from the environment and the presence of soluble bicarbonate salts, while in the case of wood iconostasis increases compared to normal 10–12% of dry wood due to woods ability to quickly absorb excess water from the air and release it by acting as a buffer material, keeping the RH approximately constant in space. Having all that in mind, biofilm and patina development on stone, and fungal proliferation on fresco and iconostasis surfaces, as well as high values (5.3 to 6.4; danger zone) documented with rapid ATP bioluminescence method on all sampling points are to be expected. 

Culturable mycobiome obtained from various substrata within Cave Church of Sts. Peter and Paul are in accordance with other mycological studies performed in caves and hypogean environments, with similar humidity and temperature conditions, indicating that these fungi have a particular affinity for environmental parameters prevailing in this type of cultural heritage site. The majority of documented culturable fungi have already been observed on these types of substrata (fresco, wood, and cave stone walls) in similar studies [55,56,57,58,59,60,61,62]. Several fungal species were isolated for the first time (e.g., *Alternaria abundans*, *Penicillium freii,* and *P. samnosnianum* from fresco; *Aspergillus aureolatus* and *Blastobotrys niveus* from iconostasis; *As. pseudoglaucus*, *Beauveria pseudobassiana*, *Fusarium sporotrichioides*, *Mortierella alpina,* and *P. expansum* from cave stone); however, fungi from these genera are known to colonize or be deposited on the surface of these cultural heritage substrata. In the case of two *Basidiomycota* isolated from wooden iconostasis (*Coprinellus disseminatus* and *Stereum gausapatum*), to the best of our knowledge, this is the first time they were documented on wooden substrata within cave churches. In the case of *S. gausapatum*, numerous fully developed, dry, fruiting bodies were observed on the basis of the iconostasis on torn-off wooden fragments. Fruiting bodies of representatives of these two genera were observed before on wood drainpipes of the Latvian Ethnographic open-air museum (*S. sanguinolentum*) and wooden chairs, benches, and barrel vaults that are part of the Republic of North Macedonia cultural heritage collection (*S. hirsutum*) [63,64] indicating that if environmental conditions are favorable and objects neglected wood-decay fungi will fructificate. Furthermore, the isolation of *Botryotrichum murorum* (syn. *Chaetomium murorum*), though previously unreported from iconostasis, is not unexpected due to its pronounced cellulolytic activities. It has been known to occur frequently in documentary heritage, frescos, and stone surfaces [65,66].

One of the more interesting fungal species, isolated from the studied deteriorated fresco, is hyphomycetes *Parengyodontium album*, commonly reported on fresco paintings in religious monuments such as churches, cathedrals, or monasteries, but also from stone walls and fresco paintings within various caves of cultural interest [67]. The potential of this fungus to colonize fresco paintings is attributed to its ability to utilize products used in the restoration of frescoes, as well as synthetic resins, calcium caseinate, and liquifying animal glue as nutrient sources. *Parengyodontium album* has been associated with several different deterioration symptoms: salt efflorescence is the most frequent, followed by discoloration and patinas of various colors, e.g., pink, ochre, dark/black, and green–black [67]. 

In vitro, biodeterioration plate assays demonstrated the potential of isolated fungi to degrade fresco, stone, and wood via the production of acids and enzymes, and to affect these substrata aesthetically, by the production of extracellular pigments. Acid metabolite production is regarded as one of the most important mechanisms of the chemical deterioration of cultural heritage and it has been claimed that most fungal species possess the ability to excrete organic acids [68]. One-quarter of tested fungi, especially those isolated from fresco surface, demonstrated positive reactions on CREA medium, indicating their potential to produce acidic metabolites. The fact that acid production is observed in a great number of isolates is consistent with the results reported earlier by the authors using the same biodegradation assays [16,30]. Namely, Savković et al. [30] demonstrated acid production for the majority of airborne *Aspergillus* and *Penicillium* isolates from cultural heritage conservation premises, while Unković et al. [16] demonstrated this ability for *Cladosporium cladosporioides*, and several *Aspergillus* and *Penicillium* isolates from sacral ambient.

*Cladosporium cladosporioides* and *P. samnosnianum*, both isolated from fresco surface, are the only two species for whom the carbonate dissolution, the primary cause of structural alterations of carbonate substrata, was documented. Various research conducted up to date, with fungi obtained from contaminated cultural heritage objects, demonstrated carbonate dissolution ability for only a small number of fungi, mainly of *Aspergillus* and *Penicillium* genera [16,33,69]. On the other hand, to the best of our knowledge, this is the first time that carbonate dissolution ability was observed for fungi of the genus *Cladosporium*, i.e., *C. cladosporioides.* In the case of *P. samnosnianum*, where a substantial reduction of pH values was documented in the acid production test (pH 4.75), carbonate dissolution probably occurred through the secretion of organic acids (with oxalic acid in most instances) that dissolve calcium carbonate, while *C. cladosporioides* could utilize one of several proposed mechanisms, such as enzymatic dissolution, ligand activity, and oxidation-reduction of redox-sensitive elements [16]. Trovão et al. [66] demonstrated carbonate dissolution in vitro for *Pa. album* and several *Penicillium* species isolated from walls of Coimbra cathedral in Portugal. 

In proteolytic activity assay, the ability to degrade casein was documented in fungi of genera *Penicillium*, *Parengyodontium*, *Cladosporium,* and *Bjerkandera*, all isolated from the painted layer of studied fresco. The potential of *C. cladosporioides* and *Bj. adusta* to degrade proteinaceous substrate of cultural heritage objects and subsequently cause irreversible damages, as well as *Parengyodontium album,* to act as a causative agent of opportunistic mycoses, was previously reported by Macêdo et al. [70], Pangallo et al. [33] and Unković et al. [16]. However, this seems to be the first record of *P. samsonianum* and *P. freii* having proteolytic activity and posing threat to wall paintings where mineral pigments were bound with casein.

Extracellular pigment production was observed for one-fifth of tested fungi with the coloration of media mostly in some hue of yellow. Pigment production by *Aspergillus* and *Penicillium* species is well known and documented by other authors: Borrego et al. [71], Unković et al. [16], Savković et al. [30], to name just a few. To the best of our knowledge, the yellow pigment was not reported in *Be. pseudobassiana*, although Ávila-Hernández et al. [72] reported a yellow pigment in a closely related *Be. bassiana*. Conversely, yellowish pigments were documented for *S. gausapatum* by Boddy and Reiner [73]. Pigment production by fungal dwellers of cultural heritage is of great importance since it leads to, usually hard if not impossible to remove, discolorations and aesthetic changes in the original artwork. It should be noted that the growth of melanized fungi, such as *Cladosporium* and *Alternaria* species documented in our study with negative reactions in pigment production assay, can nonetheless lead to the appearance of observable dark colorations of cultural heritage objects which are notoriously hard to remove due to pigment melanin [17]. In the work of Caneva et al. [4] gray patina was characterized by the growth of *C. sphaerospermum* and meristematic fungi that paralleled the reduction in humidity levels and the increase in the amounts of organic substances, probably derived from the decay of pre-existing organisms.

The dominant presence of fungi from phylum *Ascomycota*, and to a lesser extent phylum *Basidiomycota*, observed in the study by metagenomic analysis, was also documented using culture-independent techniques on Palaeolithic representations in the Escoural Cave (Alentejo, Portugal) [74]. A high abundance of the lichen-forming and rock-inhabiting fungi from class *Dothideomycetes* (mainly of order *Capnodiales*), as well as the lower but almost consistent presence of class *Agaricomycetes*, across all samples, was shown to be consistent with the literature and was already reported on limestone walls of the Church of São Salvador da Trofa in the Portuguese district of Aveiro [9]. The abundant presence of entomopathogenic fungi, such as the one from order *Lecanicillium*, on fresco paintings and cave walls may suggest that insects act as vectors in fungal dispersion to and from the walls within the cave [75] thus contributing to the colonization of the painted layer of fresco paintings.

Moderately high RA of *Pseudostichococcus monallantoides* (*Chlorophyta*) was detected in a green patina observed on a stone wall near the fresco. This green alga, previously known as a colonizer of stone relief within the hypogean heritage site of Pommery Champagne cellar [76], is phycobiont of lichenized fungus *Verrucaria* sp., documented with moderately high RA as well, and is known to induce biopitting in limestone [77] such as the one observed on limestone walls of the investigated cave church. Furthermore, among different positive correlations observed between taxa in co-occurrence analysis, the most intriguing positive interaction is the one between the lichenized fungus *Verrucaria* and mushroom-forming fungus *Coprinellus*, indicating that the presence of one always entails the presence of the other. Positive interaction between these two distinct ecological groups is quite unexpected given that *Verrucaria* is stone-inhabiting lichen, while *Coprinellus* is most likely present in form of airborne basidiospores attached to stone and lichen surfaces with an unclear role in stone colonization [78]. The presence of *Coprinellus* was previously reported to always be associated with black discoloration of limestone, which we presume to be from crustose lichen thalli, though the origin of this alteration was not discerned [9]. A fungal mat resembling a pinkish-gray powdery surface, a dominant deterioration symptom on the wooden iconostasis, was proved to be formed by a member of the *Hypoxylon* genus which was expected as they are most commonly encountered ascomycetes group on wood in the temperate regions and usually one of the earliest fungi to colonize dead wood [79]. The dominant presence of *Hypoxylon fuscopurpureum* with a relative abundance of 78.5%, identified via metabarcoding analysis, was confirmed by morphological traits observed and analyzed using in situ, optical, and SEM analysis. Conclusively, this fungus can be regarded as the main biodeterioration agent of the studied wooden iconostasis, and members of this genus are known to induce soft rot of wood used architecturally often in contact with soil and in dump conditions as one that exists in churches and hypogean environments [80]. 

Microscopic analysis of samples taken from the dominant deterioration symptom on studied fresco, i.e., wet grayish discolorations of variable size and form, have revealed reproductive structures of Cladosporium-like fungus. Metabarcoding analysis of the same sample has revealed the predominant presence of *Capnodiales* most likely from genera *Annellosympodiella*, *Neodevriesia*, and *Catenulostroma.* The morphological characteristics of the observed reproductive structures showed the greatest similarity with the *Neodevriesia* genus. Fungi from this genus were previously reported using the culture-independent method, as the dominant group on 19th century mural paintings at Tha Kham temple in Thailand [18]. This genus, recently segregated from *Cladosporium*, encompasses species usually referred to as Cladosporium-like and of variable ecological preferences including extremophiles [81]. Members of the *Neodevriesia* genus include rock-inhabitant black fungi, characterized as highly melanized and slow-growing organisms exceptionally skilled at exploiting most kinds of extreme environments [9]. As was suggested by Suphaphimol et al. [18] fungi of the *Neodevriesia* genus probably originated from the surrounding stone walls which is most likely the case of fresco deterioration in our investigation as well since RA of this fungus, recognized as *Capnodiales* (unclassified *Mycosphaerellaceae*) in metabarcoding analysis, is up to 58.5% on stone walls around fresco. 

The findings of this study have to be seen in light of some limitations. First, within the cave church, there are several substrata that have not been analyzed, such as concrete and stone as part of the entrance wall and altar area of the church, since the main focus of the research was to analyze substrata with visible symptoms of deterioration. Further research will include these substrata to obtain complete insight into the fungal diversity within the cave church. Additionally, some of the limitations might arise from the NGS sequencing short read results and could potentially lead to a lack of awareness of species levels, particularly those with the highest relative abundance. However, combining different approaches can improve and confirm our findings, as we presented in the current study. Moreover, in the future, some of the more sophisticated NGS techniques will be performed and precise information will be obtained.

## 5. Conclusions

This research gives insight into the problem of identifying fungi that are the main deterioration agents of cultural heritage objects. As a rule, extremophilic fungi that actively grow on frescoes and other works of art cannot be isolated. It is well known that the synergy of data obtained by applying both traditional and DNA-based methods gives the most precise identification of fungi and is of equal importance. The application of integrative methods, especially cross-referencing of total fungal diversity obtained by metabarcoding analysis with the morphological identification of the deterioration agents singled out and enabled the precise identification of fungi that grow actively on the “The Bald-headed Jesus’’ fresco (*Neodevriesia* sp.) and the wooden iconostasis (*H. fuscopurpureum*) within the Cave Church of Sts. Peter and Paul in Rsovci, Serbia. The metabarcoding analysis provides insight into the total mycobiome of the investigated substrate and directs the selection from the identified taxa to the few that possess the highest relative abundance and are simultaneously detected via microscopic methods as actively growing and causing deterioration. In this way, the results of the metabarcoding analysis allow us to focus on certain taxa and using appropriate identification keys and other available literature definitely identify them as the main agents of deterioration. Together, the traditional identification of fungi based on morphological features and modern DNA-based methods are powerful tools for accurate identification, especially of extremophiles as causative agents of deterioration of cultural heritage objects. This combined approach contributes to the development of strategies for conservation since it allows us to, based on the knowledge of the biology of the species, select appropriate nutrient media to isolate and cultivate the agents of deterioration, characterize their deterioration potential, and finally test their sensitivity to fungicides applicable in conservation, so conservators can select the appropriate procedures and biocides.

## Figures and Tables

**Table 1:** Zone of cleanliness and material moisture content (%) values of various sampling points on cave walls, fresco, and iconostasis within Cave Church of Sts. Peter and Paul.

Sampling Point	Zone of Cleanliness	Material Moisture Content (%)
Sample 01—depiction of Jesus	5.4—Danger zone	2.2
Sample 02—entire fresco except depiction of Jesus	5.6—Danger zone	3.0
Sample 03—gray discoloration on fresco	5.4—Danger zone	1.6
Samples 04–05—fresco surface damage	5.3 – Danger zone	1.7
Sample 06—pinkish gray powdery surface of iconostasis	6.4—Danger zone	17.1
Sample 07—blue-green deposits on stone wall under the fresco	6.1—Danger zone	20.1
Sample 08—green patina on stone wall	5.7—Danger zone	23.6
Sample 09—black deposits on stone wall	5.5—Danger zone	23.8
Sample 10—white deposits on stone wall	5.3—Danger zone	22.4
Sample 11—pink deposits on stone wall	5.5—Danger zone	22.0

**Table 2:** Biodegradative profile of culturable fungi from the Cave Church of Sts. Peter and Paul.

No.	Strain	Isolate	Origin	Biodegradation Activity
1	BEOFB218m	*Alternaria alternata*	Iconostasis	CA, HA, LA (Lac, LiP, MnP)
2	BEOFB3320m	*Aspergillus aureolatus*		LA (Lac, MnP), PP (#9b9400)
3	BEOFB6100m	*Blastobotrys niveus*		-
4	BEOFB5701m	*Botryotrichum murorum*		CA, HA, LA (Lac, LiP, MnP)
5	BEOFB18212m	*Cladosporium cladosporioides*		-
6	BEOFB2120	*Coprinellus disseminatus*		AP (6.73), HA, LA (Lac, LiP, MnP)
7	BEOFB11191m	*Penicillium citreonigrum*		LA (Lac, MnP)
8	BEOFB1730	*Stereum gausapatum*		CA, HA, LA (Lac, MnP), PP (#eaa221)
9	BEOFB280m	*Alternaria abundans*	Fresco	-
10	BEOFB290m	*Alternaria brassicae*		-
11	BEOFB1605	*Bjerkandera adusta*		AP (6.65), PA, PP (#beb72e)
12	BEOFB3105m	*Botrytis cinerea*		AP (4.80)
13	BEOFB18216m	*Cladosporium cladosporioides*		AP (6.72), CD, PA
14	BEOFB6001m	*Mortierella alpina*		-
15	BEOFB6200m	*Parengyodontium album*		PA
16	BEOFB1109m	*Penicillium brevicompactum*		-
17	BEOFB11230m	*Penicillium frei*		AP (4.27), PA, PP (#8db600)
18	BEOFB11211m	*Penicillium samsonianum*		AP (4.75), CD, PA
19	BEOFB219m	*Alternaria alternata*	Stone	-
20	BEOFB3321m	*Aspergillus aureolatus*		PP (#9b9400)
21	BEOFB3174m	*Aspergillus pseudoglaucus*		-
22	BEOFB2910m	*Beauveria pseudobassiana*		PP (#e9e450)
23	BEOFB18219m	*Cladosporium cladosporioides*		-
24	BEOFB2130	*Coprinellus domesticus*		-
25	BEOFB1711m	*Epicoccum nigrum*		-
26	BEOFB863m	*Fusarium sporotrichioides*		-
27	BEOFB6006m	*Mortierella alpina*		AP (4.90)
28	BEOFB3511m	*Mucor hiemalis*		-
29	BEOFB11132m	*Penicillium expansum*		AP (6.72)
30	BEOFB1260m	*Trichoderma viridescens*		-

AP—acid production (documented pH values); PA—proteolytic activity; CA—cellulolytic activity; HA—hemicellulolytic activity; LA—ligninolytic activity (Lac—laccase, LiP—lignin peroxidase, MnP—Mn-oxidizing peroxidases); CD—carbonate dissolution; PP—pigment production (NBS-ISCC color hex code).

**Table 3:** Alpha diversity within the analyzed samples from fresco and around it (average values) and presented at the phylum, family, genus, and ASV taxa levels.

Indices	Description	Mean	St. Dev.	Min.	Max.	Kruskal–Wallis Sign. (*p* < 0.05)
Phylum						
OBS	Fresco	3.00	0.00	3.00	3.00	0.224
	Around Fresco	3.67	1.21	3.00	6.00	
Chao1	Fresco	3.00	0.00	3.00	3.00	0.224
	Around Fresco	3.67	1.21	3.00	6.00	
ACE	Fresco	3.00	0.00	3.00	3.00	0.224
	Around Fresco	3.67	1.21	3.00	6.00	
Shannon	Fresco	0.11	0.09	0.02	0.22	0.019
	Around Fresco	0.34	0.13	0.16	0.52	
Gini–Simpson	Fresco	0.05	0.04	0.01	0.11	0.019
	Around Fresco	0.17	0.09	0.06	0.32	
Family						
OBS	Fresco	56.75	29.88	22.00	95.00	0.136
	Around Fresco	109.50	43.16	45.00	153.00	
Chao1	Fresco	57.65	30.14	22.00	95.75	0.136
	Around Fresco	110.77	44.09	45.20	157.09	
ACE	Fresco	57.88	30.13	22.23	95.96	0.136
	Around Fresco	111.45	43.77	46.08	157.69	
Shannon	Fresco	1.17	0.33	0.78	1.58	0.088
	Around Fresco	2.11	0.86	1.08	3.24	
Gini–Simpson	Fresco	0.49	0.06	0.42	0.56	0.088
	Around Fresco	0.72	0.21	0.38	0.92	
Genus						
OBS	Fresco	90.00	61.07	26.00	173.00	0.136
	Around Fresco	181.17	91.37	60.00	276.00	
Chao1	Fresco	93.43	61.44	26.00	175.10	0.136
	Around Fresco	184.92	91.94	62.50	285.05	
ACE	Fresco	92.86	61.53	26.19	175.25	0.136
	Around Fresco	185.07	90.79	64.38	283.09	
Shannon	Fresco	1.22	0.37	0.78	1.69	0.088
	Around Fresco	2.28	1.02	1.10	3.55	
Gini–Simpson	Fresco	0.49	0.06	0.42	0.56	0.088
	Around Fresco	0.73	0.21	0.38	0.93	
ASV						
OBS	Fresco	155.50	108.82	47.00	306.00	0.088
	Around Fresco	340.00	169.06	141.00	526.00	
Chao1	Fresco	158.40	110.29	47.00	310.50	0.088
	Around Fresco	349.57	171.66	141.86	551.60	
ACE	Fresco	157.79	109.58	47.00	308.76	0.088
	Around Fresco	348.55	171.91	142.27	551.26	
Shannon	Fresco	2.08	0.40	1.58	2.54	0.201
	Around Fresco	3.09	1.12	1.70	4.32	
Gini–Simpson	Fresco	0.73	0.04	0.67	0.78	0.286
	Around Fresco	0.85	0.14	0.64	0.97	

**Table 4:** Differential abundance analysis of statistically significant species according to the origin of the samples.

Taxon Name	baseMean	log2 FoldChange	lfcSE	Stat	*p* Value
*Hirsutella*	711.943552	−10.86822846	1.941390071	−4.567978681	4.9245 × 10^−6^
*Pyrenochaeta*	37.8113517	21.52668696	2.644391638	7.384188741	1.53386 × 10^−13^
*Phialophora*	101.742202	22.76808504	2.77610541	7.481014576	7.3751 × 10^−14^
*Rhinocladiella*	166.739135	9.386290822	2.156576309	3.425007867	0.000614782
**Taxon Name**	***p* adj**	**log2 FoldChange_apeglm**	**lfcSE_apeglm**	***p* value_apeglm**	***p* adj_apeglm**
*Hirsutella*	0.000428432	−10.2402688	1.941237778	2.16628 × 10^−8^	1.69692 × 10^−6^
*Pyrenochaeta*	2.00168 × 10^−11^	0.46897268	1.186974431	3.93628 × 10^−16^	4.62513 × 10^−14^
*Phialophora*	1.9249 × 10^−11^	0.4200758	1.154011635	2.37508 × 10^−16^	4.62513 × 10^−14^
*Rhinocladiella*	0.040114501	10.2864502	4.084514356	1.34653 × 10^−5^	0.000791086

*p*-adjusted values < 0.05 according to Benjamini and Holceberg method were considered significant.

## Data Availability

All data were deposited within the NCBI database as BioProject ID: PRJNA826742 (under the accessions from SAMN27582357 to SAMN27582366).

## Supplementary Materials

The following supporting information can be downloaded at: https://www.mdpi.com/article/10.3390/jof8121263/s1, Figure S1: Rarefaction analysis at the ASV, genus, family, and phylum taxa levels; Figure S2: The DPCoA plot created with Bray–Curtis dissimilarities at the ASV, genus, family and phylum taxa levels, to present the variability within and between groups of samples; Table S1: Number of the read retention and sequence decontamination; Table S2: Molecular analyses of culturable fungi from the Cave Church of Sts. Peter and Paul; Table S3: Alpha diversity within the analyzed individual samples from fresco painting and from sampling points of the cave interior and presented at the phylum, family, genus, and ASV level.
