# Supplementary material for: Mycobiome Diversity of the Cave Church of Sts. Peter and Paul in Serbia—Risk Assessment Implication for the Conservation of Rare Cavern Habitat Housing a Peculiar Fresco Painting
Source: J Fungi (Basel). 2022 Nov 30;8(12):1263. doi: 10.3390/jof8121263 (PMC9782640; doi:10.3390/jof8121263)
Supplement: Supplementary file 1 [file jof-08-01263-s001.zip › Supplementary Table S1.pdf]

Table S1: Number of the read retention and sequence decontamination.

Sample	Input	Primer remove	Quality filter	DenoisedF	DenoisedR	Merged	Chimera remove	Length trim	Decontaminated reads	Percent retained	Final % of reads
01	146032	146011	126074	125920	125926	123833	123243	123123	123123	100.00	84.31
02	121155	121129	105719	105598	105520	103775	103366	103143	103132	99.99	85.12
03	131888	131489	115908	115782	115749	114170	113671	113529	113529	100.00	86.08
04-05	144142	144109	124385	123796	123816	120118	119639	119189	119030	99.87	82.58
06	222761	222756	195332	195034	195046	194118	190250	190146	190144	100.00	85.36
07	113986	113400	95391	94974	94847	85837	85470	76784	76734	99.93	67.32
08	195373	195332	165480	164280	164969	158680	157365	156531	156531	100.00	80.12
09	106494	106142	92015	91465	91529	88405	87916	86065	86065	100.00	80.82
10	124493	124422	104819	104293	104341	101754	101204	97881	97746	99.86	78.52
11	198895	198864	171957	171183	171198	165199	162917	161419	161382	99.98	81.14
Control	123	83	15	10	10	10	10	10	10	-	8.13
