# Supplementary material for: Mycobiome Diversity of the Cave Church of Sts. Peter and Paul in Serbia—Risk Assessment Implication for the Conservation of Rare Cavern Habitat Housing a Peculiar Fresco Painting
Source: J Fungi (Basel). 2022 Nov 30;8(12):1263. doi: 10.3390/jof8121263 (PMC9782640; doi:10.3390/jof8121263)
Supplement: Supplementary file 1 [file jof-08-01263-s001.zip › Supplementary Table S2.pdf]

Supplementary Table S2. Molecular analyses of culturable fungi from the cave Church of Sts. Peter and Paul

No	Strain	Isolate	Gene bank accession number		Homology	
			ITS	*BenA*	*ITS*	*BenA*
1	BEOFB280m	*Alternaria abundans*	OL457279	-	100.00	-
2	BEOFB218m	*Alternaria alternata*	OL457258	-	100.00	-
3	BEOFB219m	*Alternaria alternata*	OL457273	-	99.81	-
4	BEOFB2110m	*Alternaria alternata*	OL457275	-	99.81	-
5	BEOFB290m	*Alternaria brassicae*	OL457269	-	99.81	-
6	BEOFB3320m	*Aspergillus aureolatus*	OL457263	OL631576	99.44	100.00
7	BEOFB3321m	*Aspergillus aureolatus*	OL457292	OL631583	99.81	100.00
8	BEOFB3174m	*Aspergillus pseudoglaucus*	OL457285	OL631581	100.00	100.00
9	BEOFB2910m	*Beauveria pseudobassiana*	OL457286	-	99.43	-
10	BEOFB1605	*Bjerkandera adusta*	OL457268	-	100.00	-
11	BEOFB6100m	*Blastobotrys niveus*	OL405446	-	89.54	-
12	BEOFB5701m	*Botryotrichum murorum*	OL405554	-	99.81	-
13	BEOFB3105m	*Botrytis cinerea*	OL457283	-	100.00	-
14	BEOFB18212m	*Cladosporium cladosporioides*	OL457259	-	100.00	-
15	BEOFB18213m	*Cladosporium cladosporioides*	OL457260	-	100.00	-
16	BEOFB18214m	*Cladosporium cladosporioides*	OL457262	-	100.00	-
17	BEOFB18215m	*Cladosporium cladosporioides*	OL457266	-	99.80	-
18	BEOFB18216m	*Cladosporium cladosporioides*	OL457267	-	100.00	-
19	BEOFB18217m	*Cladosporium cladosporioides*	OL457278	-	100.00	-
20	BEOFB18218m	*Cladosporium cladosporioides*	OL457282	-	100.00	-
21	BEOFB18219m	*Cladosporium cladosporioides*	OL457288	-	99.38	-
22	BEOFB2120	*Coprinellus disseminatus*	OL457261	-	99.85	-
23	BEOFB2130	*Coprinellus domesticus*	OL457272	-	99.29	-
24	BEOFB1711m	*Epicoccum nigrum*	OL457274	-	96.97	-
25	BEOFB863m	*Fusarium sporotrichioides*	OL457287	OL631582	100.00	100.00
26	BEOFB6001m	*Mortierella alpina*	OL457284	-	99.84	-
27	BEOFB6002m	*Mortierella alpina*	OL457289	-	99.68	-
28	BEOFB6003m	*Mortierella alpina*	OL457291	-	99.68	-
29	BEOFB6004m	*Mortierella alpina*	OL457293	-	99.67	-
30	BEOFB6005m	*Mortierella alpina*	OL457294	-	99.68	-
31	BEOFB6006m	*Mortierella alpina*	OL457270	-	99.84	-
32	BEOFB3511m	*Mucor hiemalis*	OL457271	-	99.84	-
33	BEOFB6200m	*Parengyodontium album*	OL457280	-	100.00	-
34	BEOFB1109m	*Penicillium brevicompactum*	OL457281	OL631580	99.81	100.00
35	BEOFB11191m	*Penicillium citreonigrum*	OL405444	OL519900	99.62	100.00
36	BEOFB11192m	*Penicillium citreonigrum*	OL405565	OL519901	100.00	100.00
37	BEOFB11193m	*Penicillium citreonigrum*	OL457264	OL631577	100.00	100.00
38	BEOFB11132m	*Penicillium expansum*	OL457295	OL631584	100.00	100.00
39	BEOFB11230m	*Penicillium frei*	OL457277	OL631579	99.63	100.00
40	BEOFB11211m	*Penicillium samsonianum*	OL457265	OL631578	99.62	100.00
41	BEOFB1730	*Stereum gausapatum*	OL457276	-	100.00	-
42	BEOFB1260m	*Trichoderma viridescens*	OL457290	-	99.82	-
