# Supplementary material for: Mycobiome Diversity of the Cave Church of Sts. Peter and Paul in Serbia—Risk Assessment Implication for the Conservation of Rare Cavern Habitat Housing a Peculiar Fresco Painting
Source: J Fungi (Basel). 2022 Nov 30;8(12):1263. doi: 10.3390/jof8121263 (PMC9782640; doi:10.3390/jof8121263)
Supplement: Supplementary file 1 [file jof-08-01263-s001.zip › Supplementary Table S3.pdf]

Table S3: Alpha diversity within the analyzed individual samples from fresco painting and from sampling points of the cave interior and presented at the phylum, family, genus, and ASV level.

Sample	Description	OBS	Chao1	se.chao1	ACE	se.ACE	Shannon	Gini-Simpson	Inv Simpson	level
01	Depiction of Jesus	124	130.4	5.5	129.0	5.3	2.0	0.7	3.8	ASV
02	Entire fresco except depiction of Jesus	145	145.7	1.1	146.4	5.7	2.2	0.8	4.0	
03	Gray discoloration on fresco	47	47.0	0.0	47.0	3.1	1.6	0.7	3.1	
04-05	Fresco surface damage	306	310.5	3.9	308.8	8.4	2.5	0.8	4.5	
06	Iconostasis	141	141.9	1.4	142.3	5.7	1.7	0.6	2.8	
07	Blue-green deposits on stone wall under the fresco	421	422.9	2.3	422.4	8.3	4.3	1.0	30.3	
08	Green patina on stone wall	183	207.0	11.0	202.4	6.7	1.8	0.7	3.5	
09	Black deposits on stone wall	255	255.0	0.1	255.2	6.4	3.6	0.9	15.9	
10	White deposits on stone wall	514	519.1	3.8	517.6	10.9	4.0	1.0	21.3	
11	Pink deposits on stone wall	526	551.6	9.2	551.3	11.3	3.2	0.9	10.8	
01	Depiction of Jesus	77	88.0	8.9	85.1	4.4	1.2	0.5	1.9	Genus
02	Entire fresco except depiction of Jesus	84	84.6	1.2	84.9	4.3	1.3	0.5	2.0	
03	Gray discoloration on fresco	26	26.0	0.5	26.2	2.5	0.8	0.4	1.7	
04-05	Fresco surface damage	173	175.1	2.3	175.3	6.5	1.7	0.6	2.3	
06	Iconostasis	60	62.5	2.9	64.4	3.8	1.1	0.4	1.6	
07	Blue-green deposits on stone wall under the fresco	235	235.3	0.9	235.5	5.9	3.5	0.9	14.6	
08	Green patina on stone wall	112	119.6	5.0	120.7	5.1	1.3	0.6	2.8	
09	Black deposits on stone wall	133	133.8	1.4	133.8	4.9	2.6	0.9	7.0	
10	White deposits on stone wall	271	273.3	2.6	273.0	7.3	3.3	0.9	12.2	
11	Pink deposits on stone wall	276	285.0	5.5	283.1	8.2	1.8	0.6	2.7	
01	Depiction of Jesus	54	56.5	3.2	56.6	3.6	1.1	0.5	1.9	Family
02	Entire fresco except depiction of Jesus	56	56.3	0.9	56.7	3.5	1.2	0.5	2.0	
03	Gray discoloration on fresco	22	22.0	0.5	22.2	2.2	0.8	0.4	1.7	
04-05	Fresco surface damage	95	95.8	1.4	96.0	4.4	1.6	0.6	2.3	
06	Iconostasis	45	45.2	0.6	46.1	3.1	1.1	0.4	1.6	
07	Blue-green deposits on stone wall under the fresco	138	139.0	1.8	139.2	3.7	3.2	0.9	13.1	
08	Green patina on stone	83	84.3	1.6	86.2	4.4	1.3	0.6	2.8	

	wall									
09	Black deposits on stone wall	91	91.0	0.1	91.3	3.2	2.5	0.9	6.9	
10	White deposits on stone wall	147	148.0	1.6	148.2	5.4	2.8	0.9	8.8	
11	Pink deposits on stone wall	153	157.1	3.6	157.7	6.0	1.8	0.6	2.7	
01	Depiction of Jesus	3	3.0	0.0	3.0	0.0	0.0	0.0	1.0	Phylum
02	Entire fresco except depiction of Jesus	3	3.0	0.0	3.0	0.0	0.1	0.0	1.0	
03	Gray discoloration on fresco	3	3.0	0.0	3.0	0.0	0.1	0.1	1.1	
04-05	Fresco surface damage	3	3.0	0.0	3.0	0.0	0.2	0.1	1.1	
06	Iconostasis	3	3.0	0.0	3.0	0.0	0.2	0.1	1.1	
07	Blue-green deposits on stone wall under the fresco	6	6.0	0.0	6.0	1.2	0.4	0.2	1.3	
08	Green patina on stone wall	3	3.0	0.0	3.0	0.0	0.5	0.3	1.5	
09	Black deposits on stone wall	4	4.0	0.0	4.0	0.9	0.4	0.2	1.2	
10	White deposits on stone wall	3	3.0	0.0	3.0	0.0	0.3	0.2	1.2	
11	Pink deposits on stone wall	3	3.0	0.0	3.0	0.0	0.3	0.1	1.1	
