# Supplementary figures and images for: Mycobiome Diversity of the Cave Church of Sts. Peter and Paul in Serbia—Risk Assessment Implication for the Conservation of Rare Cavern Habitat Housing a Peculiar Fresco Painting
Source: J Fungi (Basel). 2022 Nov 30;8(12):1263. doi: 10.3390/jof8121263 (PMC9782640; doi:10.3390/jof8121263)

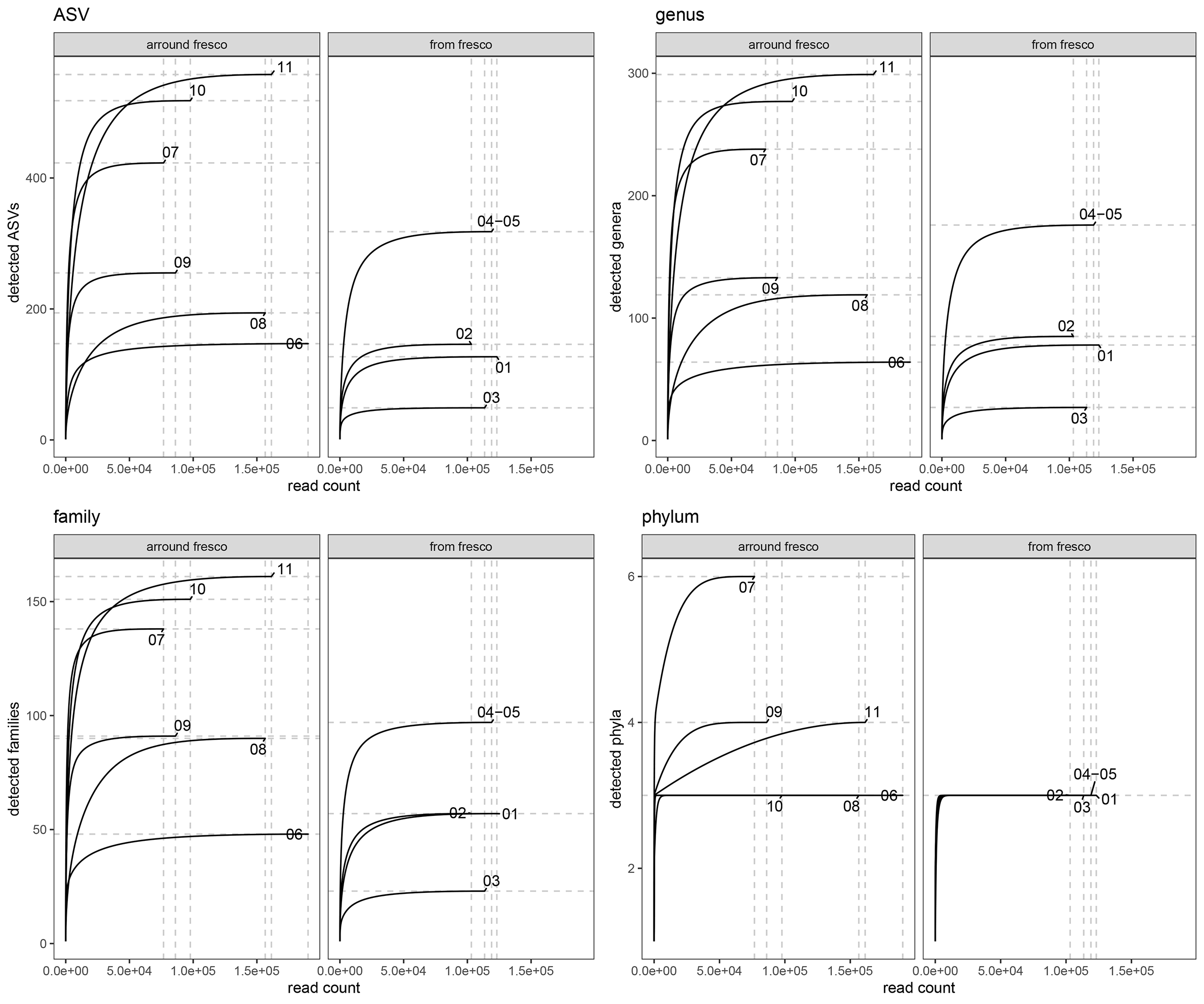

Supplement: Supplementary file 1 [file jof-08-01263-s001.zip › Supplementary Figure S1.tif]

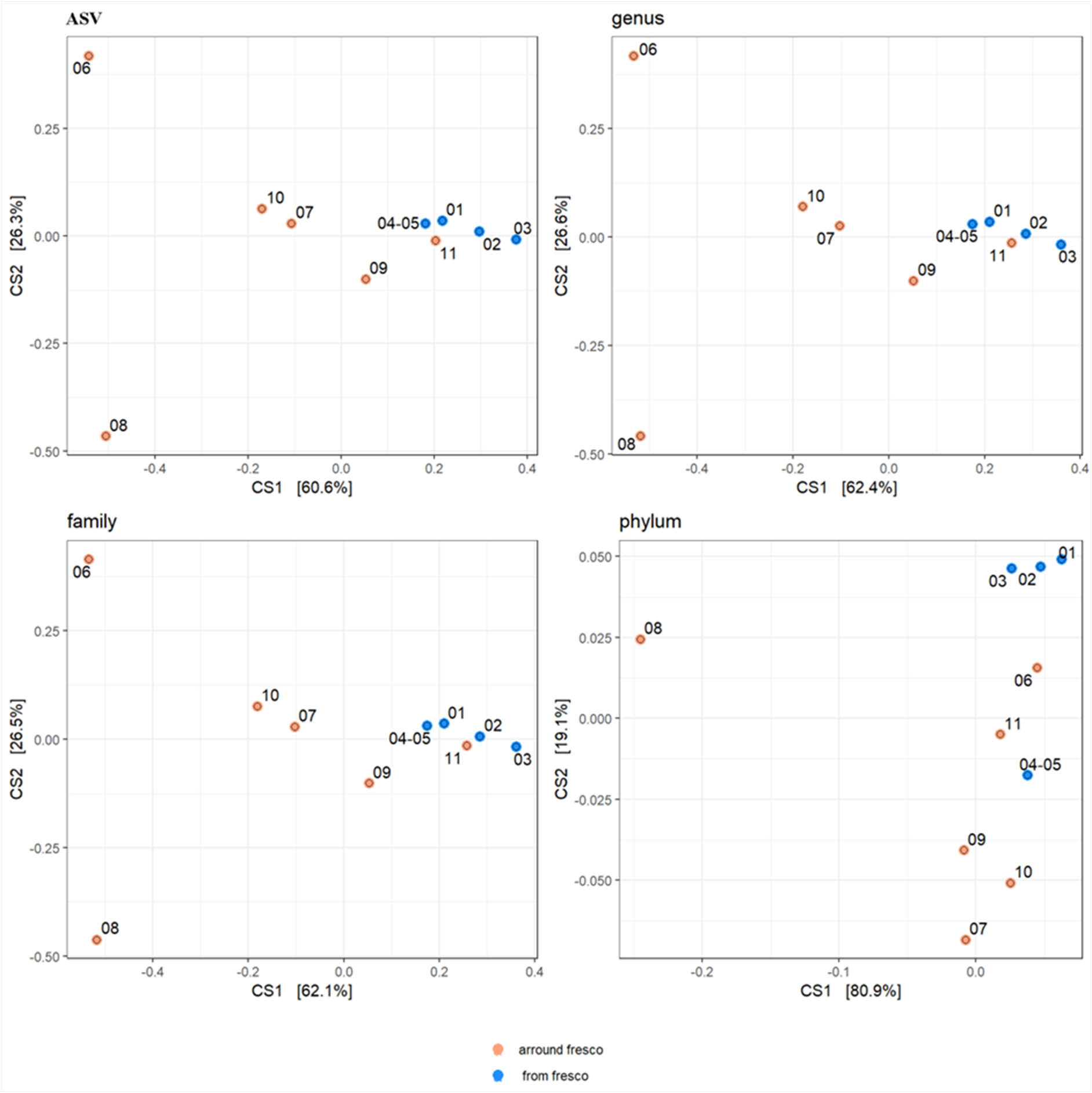

Supplement: Supplementary file 1 [file jof-08-01263-s001.zip › Supplementary Figure S2.tif]
